# Unraveling Heat Integration Opportunities in SOFC–Ethanol Reformer Systems across Steam Reforming, Partial Oxidation, and Autothermal Reforming Pathways

**DOI:** 10.1021/acsomega.5c09407

**Published:** 2025-11-18

**Authors:** Eduardo F. Beathalter, Guilherme P. Pickler, Bruno F. Oechsler, Amir A. M. Oliveira, Rafael C. Catapan

**Affiliations:** † Universidade Federal de Santa Catarina, 89219-600 Joinville, SC, Brazil; ‡ Graduate Program in Mechanical Engineering (POSMEC), 28117 Universidade Federal de Santa Catarina, 88040-900 Florianópolis, SC, Brazil; § Graduate Program in Chemical Engineering (POSENQ), Universidade Federal de Santa Catarina, 88040-900 Florianópolis, SC, Brazil; ∥ Graduate Program in Mechanical Science and Engineering (PPGECM), Universidade Federal de Santa Catarina, 89219-600 Joinville, SC, Brazil

## Abstract

Heat integration is essential to enhance the efficiency of solid oxide fuel cell (SOFC) systems coupled with ethanol reformers, being fundamental to enable their use in onboard applications and distributed power systems. However, a unified, system-level comparison of steam reforming (SR), partial oxidation (POX), and autothermal reforming (ATR) within a single modeling and analysis of heat integration opportunities has been lacking. This study addresses this gap by developing a simulation framework that combines a validated lumped SOFC model with a flowsheet simulation environment to systematically assess the thermal behavior of SOFC–reformer configurations. A design of experiments approach, based on a face-centered central composite design, was employed to generate discrete simulation data, which were subsequently analyzed using analysis of variance (ANOVA) and response surface methodology (RSM). This enabled the construction of metamodels linking key process variables such as reformer and SOFC temperatures, operating pressure, water-to-ethanol, and oxygen-to-ethanol molar ratios to system responses such as electrical efficiency and thermal duties. Results show that the SOFC consistently acts as a net heat source, producing surplus heat relative to the reformer demand, while the temperature gradient between components favors internal recovery. Conditions close to the SR regime were found to maximize the electrical efficiency (up to 36%) and minimize external heating requirements. Based on these insights, a heat exchanger network (HEN) was proposed by using the Pinch method and validated in the flowsheet simulation. The proposed HEN fully satisfied the heating requirements of the system, eliminating the need for hot utilities and improving electrical efficiency from 36 to 52%. Overall, the study demonstrates that thermally integrated SOFC–ethanol reformer systems can achieve self-sustaining operation under steady-state conditions. The proposed unified modeling framework provides new insights into the thermodynamic coupling between ethanol reformers and SOFCs, highlighting ethanol as a viable renewable fuel and advancing the design of compact, fuel-flexible energy technologies for high-efficiency, and clean power generation.

## Graphical abstract

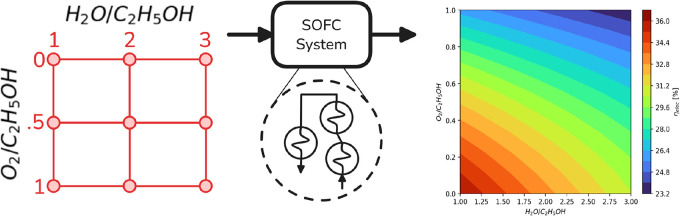

## 1 Introduction

Ethanol is emerging as a promising sustainable hydrogen source for fuel cells, since it is renewable and compatible with an existing infrastructure for production, transportation, and storage.^1^ Various technologies to produce electricity from ethanol have been explored, including direct ethanol fuel cells,^1,2^ internal combustion engine (ICE) generators,^3^ and solid oxide fuel cells (SOFCs) coupled with internal^4^ or external reformers^5^ among others. Each of these technologies, however, faces specific limitations. Direct ethanol fuel cells, although well-known by their simplicity and compactness, often suffer from low efficiency and incomplete ethanol oxidation.^1^ ICE generators exhibit relatively low electrical efficiency and contribute to significant pollutant emissions even when operated with ethanol blends.^3^ Internal reforming SOFC systems can achieve compact integration but are thermally constrained by the fuel cell temperature and susceptible to catalyst deactivation due to carbon deposition.^4^ The integration of SOFCs with an external reformer provides fuel flexibility and has the potential to enhance system efficiency by optimizing reforming conditions.

The integration of ethanol reformers with SOFC systems has been recently addressed, with a focus on specific reforming pathways or configurations. For instance, Thanomjit et al.^6^ investigated the thermodynamic performance of different ethanol reforming routes, e.g., steam reforming (SR), partial oxidation (POX), and autothermal reforming (ATR), when integrated with a solid oxide fuel cell system, providing valuable insights into the influence of operating temperature and feed composition on overall efficiency. Ma et al.^7^ modeled an SOFC system integrated with a steam reformer using ethanol as feedstock and highlighted how the water-to-ethanol molar ratio and reformer temperature affect hydrogen yield and fuel cell performance. Saebea et al.^8^ performed a thermodynamic analysis using different renewable fuels and showed that steam reforming leads to the highest hydrogen yield, while autothermal and partial oxidation routes offer better thermal efficiencies under certain conditions. Similarly, in an recent work, we proposed a heat exchanger network for thermal integration of SOFC and ethanol reformers, demonstrating that proper heat recovery could raise overall system efficiency from 44.4 to 61.2%, but still was limited to one reforming configuration per simulation case.^9^ Palus and Pianko-Oprych^10^ developed a dynamic model for a catalytic partial oxidation coupled to an SOFC and validated it experimentally, but their focus was on methane and not ethanol as a renewable fuel.

To effectively evaluate the SOFC integration within energy systems, reliable and computationally efficient models are required. Among these, zero-dimensional lumped-parameter models strike a balance between accuracy and computational cost, making them particularly suitable for integration into flowsheet process simulations.^11,12^ While they neglect detailed spatial variations, they retain essential information about system-level performance, such as power output, heat generation, and fuel requirements.^12^ These characteristics make them attractive for flowsheet-level analysis and real-time applications. However, their predictive power strongly depends on robust kinetic and transport parameters, which must be validated against experimental data.^11^ In this context, the work of Saebea et al.^8^ highlights the importance of calibration and validation steps in building reliable system models applied in SOFC systems.

Although previous studies have provided valuable insights into ethanol reforming coupled with SOFC systems, including thermodynamic analyses of multiple reforming routes and efforts toward heat integration, a comprehensive and systematic comparison of the three main ethanol reforming pathways, e.g., SR, POX, and ATR, within a unified thermodynamic and heat integration framework is still lacking to the best of the authors’ knowledge. Moreover, existing models often evaluate isolated operating conditions or simplify the thermal coupling between the reformer and the fuel cell, which limits their capacity to capture the full range of thermal interactions and performance trends across broader design spaces. Addressing this gap, the present study aims to evaluate the thermal coupling potential between SOFC and ethanol reformers by simulating all three reforming modes using a lumped SOFC model validated against experimental data and implemented within a flowsheet simulation. A metamodel is proposed to address performance parameters related to heat exchanger networks between the SOFC and the reformer over a wide range of operating conditions. At the end, a heat exchanger network is proposed to integrate the system. This approach enables a quantitative comparison of system performance and thermal coupling across reforming routes. The main contributions of this work are (i) the development and validation of a unified SOFC–reformer model suitable for system-level thermal analysis; (ii) the construction of metamodels based on response surface methodology (RSM) to identify key process interactions and optimal operating conditions; and (iii) the design and evaluation of a heat exchanger network (HEN) that enables full internal heat recovery and improves overall electrical efficiency. An overview of the methodological framework is provided in Figure 1.

**Figure 1:**
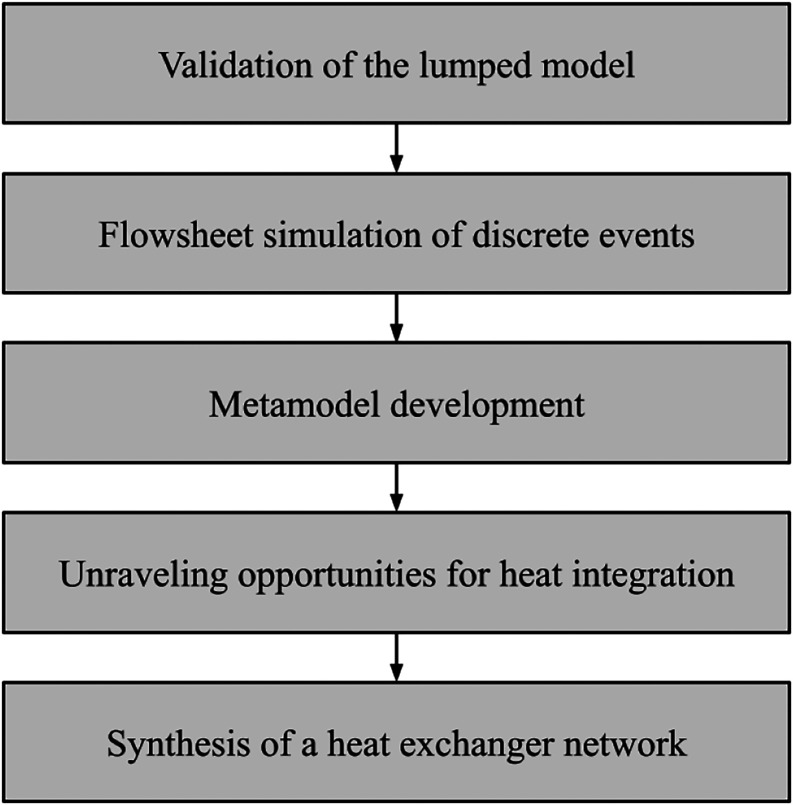
A quick overview of the methodological framework employed in the current work.

## 2 Modeling

### 2.1 Flowsheet Simulation

Flowsheet simulations using lumped SOFC models are effective for exploring optimal operating conditions without the high cost of trial-and-error experiments. DWSIM,^13^ an open-source process simulator, offers robustness, flexibility, and a reliable thermodynamic property database, and has been applied to various energy systems, such as combined cycles,^14^ solar plants,^15^ and biogas reforming.^16^

Figure 2 shows the DWSIM flowsheet developed for the SOFC–reformer system. It includes air, water, and ethanol feed lines; a reformer; an SOFC; and an afterburner. The air line has been split into three in order to provide oxygen for the reformer, SOFC, and afterburner individually. On the other hand, the water and ethanol streams have been combined before being heated and further mixed with the air for the reformer. Reforming modes (steam, partial oxidation, and autothermal) are controlled through the oxygen/ethanol and water-to-ethanol molar ratios as described further in the text. To ensure a general and flexible framework, heat exchangers were not included; instead, heaters supply the required energy to preheat all streams to user-defined reformer and SOFC temperatures. Thermodynamic properties were estimated assuming ideal fluid for single-phase streams and Raoult’s law for liquid–vapor mixed streams, suitable for nonpolar species at pressures below 10 atm.^17^

**Figure 2:**
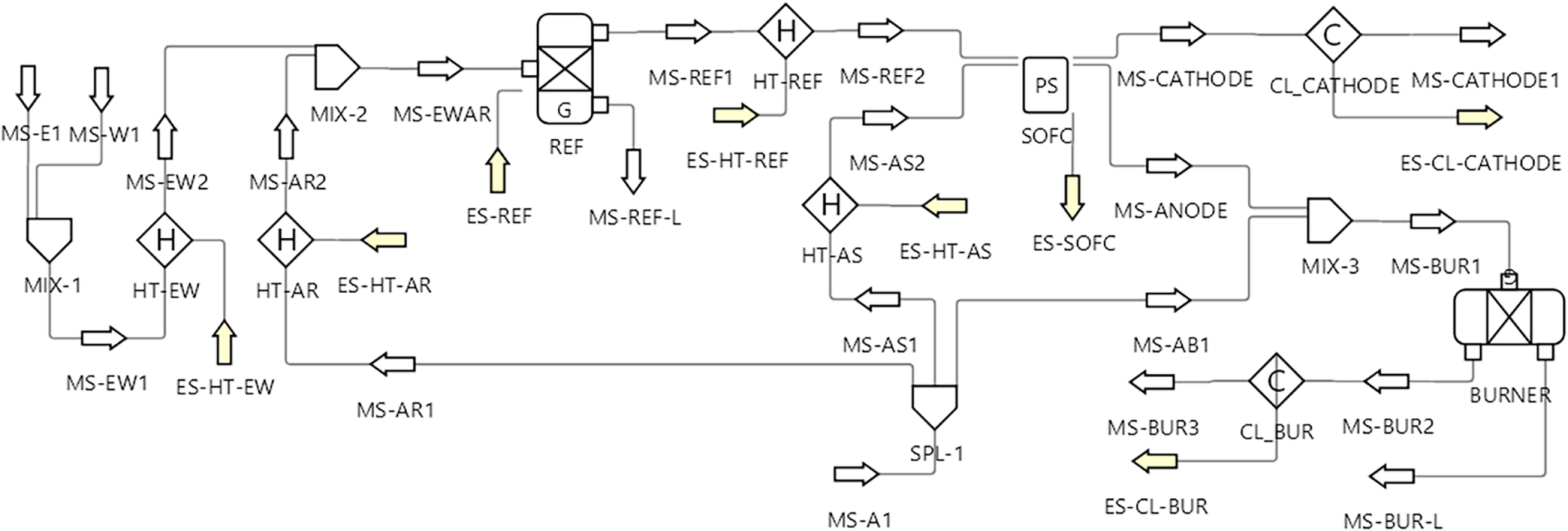
Flowsheet of the SOFC and reformer integration evaluated in DWSIM.

The system modeled in Figure 2 can be described as follows. Water and ethanol enter through MS-E1 and MS-W1, respectively. They’re mixed at MIX-1 and heated up to the reformer’s temperature at HT-EW. Similarly, air enters through MS-A1 and is then split to the reformer (MS-AR1), SOFC (MS-AS1), and afterburner (MS-AB1). Air for the reformer and SOFC are heated by HT-AR and HT-AS respectively, while air for the afterburner is not preheated. MIX-2 combines the air with ethanol and water vapors, which are then fed to the reformer (REF). An intermediary heater (HT-REF) was placed between the reformer and the SOFC to allow them to be modeled with independent temperatures. As with the reformer, the SOFC’s anode output is mixed with the afterburner air fraction by MIX-3 before entering the afterburner (BURNER). Finally, the SOFC cathode and the afterburner outlets go through coolers (CL_CATHODE and CL_BUR) in order to enable the evaluation of the leftover energy in the streams. Naturally, the energy exchange from each unit operator is denoted as the attached energy stream. The composition of the inlet material streams (MS-E1, MS-W1, and MS-A1) were kept constant at 100% C_2_H_5_OH for MS-E1, 100% H_2_O for MS-W1, and 79% N_2_/21% O_2_ for MS-A1. The ethanol, water, and air flow rates were written to their respective inlet streams, as well as the system pressure. The reformer temperatures were set as the outlet temperature of HT-EW and HT-AR, while the SOFC temperatures were applied to HT-REF and HT-AS.

It is important to note that the present analysis was conducted under steady-state assumptions. Dynamic effects such as start-up and load transients were not included as the primary goal was to identify fundamental heat integration opportunities and steady-state coupling mechanisms between the SOFC and the reformer. To evaluate the heat demand or supply across the system components, mass and energy conservation equations are applied to each unit operator, assuming steady-state operation and negligible changes in potential and kinetic energies. Under these assumptions, the conservation principles for mass (eq 1) and energy (eq 2) within a control volume are given by

1 $$\sum_{i=1}^{\mathrm{NC}}{ṁ}_{i}^{\mathrm{out}}-\sum_{i=1}^{\mathrm{NC}}{ṁ}_{i}^{\mathrm{in}}=0$$

2 $$\mathrm{Q̇}=\sum_{i}^{\mathrm{NC}}{ṁ}_{i}^{\mathrm{out}}h_{i}^{\mathrm{out}}-\sum_{i}^{\mathrm{NC}}{ṁ}_{i}^{\mathrm{in}}h_{i}^{\mathrm{in}}$$

where *ṁ*_*i*_^in^ and *ṁ*_*i*_^out^ represent the mass flow rates of the *i* ^th^ chemical species at inlets and outlets of the component, respectively, and *h*_*i*_^in^ and *h*_*i*_^out^ are the specific enthalpies at inlets and outlets, respectively. NC is the number of chemical species and streams entering the component. In this context, a component stands for a reformer, an afterburner, mixers, splitters, and heaters.

The system electrical efficiency, η_elec_, was defined as the ratio between the electricity generated by the fuel cell and the sum of the chemical energy available in the fuel and the external power supplied to the heaters. This ratio can be calculated using eq 3.

3 $$\eta_{\mathrm{elec}}=\frac{{Ṗ}_{\mathrm{SOFC}}}{{ṁ}_{\mathrm{fuel}}\cdot \mathrm{LHV}+\sum_{j}^{\mathrm{NC}}{Ṗ}_{j}}$$

where *Ṗ*_SOFC_ is the SOFC power output, *ṁ*_fuel_ is the fuel mass flow rate, LHV is the lower heating value of the fuel, *Ṗ*_*j*_ represents the power consumption by the *j* ^th^ heater, and *NC* is the total number of heaters. Specific models for the components are described in the following sections.

### 2.2 Reformer and Afterburner

The ethanol reformer follows the generalized reaction 4

R1 $$C_{2}H_{5}\mathrm{OH}+\nu O_{2}+\left(3-2\nu \right)H_{2}O\to \left(6-2\nu \right)H_{2}+2{\mathrm{CO}}_{2}$$

where *ν* denotes the stoichiometric coefficient of O_2_. When *ν* = 0, the process corresponds to steam reforming with an oxygen-to-ethanol ratio of zero and a water-to-ethanol ratio of 3. Conversely, when *ν* = 1.5, the process operates under partial oxidation with an oxygen-to-ethanol ratio of 1.5 and no steam input. Intermediate values of *ν* represent oxidative steam reforming, also known as autothermal reforming. The molar ratios of oxygen-to-ethanol and water-to-ethanol in the reformer are independently set and used as primary design variables.

Air is supplied to the afterburner to ensure a combustion equivalence ratio of 0.5 (100% air in excess) conditions, accounting for the oxidation of all possible byproducts, including, but not limited to, CO and residual H_2_. The exhaust gases of the reformer and afterburner are assumed to reach chemical equilibrium at a constant pressure. As the present work intends on assessing thermal integration possibilities between the reformer and the SOFC, the reformer has been modeled as isothermal while the afterburner has been modeled as adiabatic. Consequently, the reformer’s net energy surplus or deficit (depending on operational conditions) is written to the associated energy stream.

### 2.3 Mixers, Splitters, Heaters, and Coolers

Mixers combine multiple input streams into a single output while ensuring mass and energy balances, as governed by eqs 1 and 2, respectively. The calculation begins with the determination of the mass balance and output stream composition. Then, the pressure is set based on the user-defined configuration. Finally, the outlet temperature is computed via a PH flash calculation, using the enthalpies of the input streams.^17^ Splitters, in contrast, simply divide a single stream into multiple outputs according to user-defined flow ratios.

Heaters and coolers follow a similar computational procedure, where heat is added or removed from a stream to simulate heat exchange without the use of an auxiliary hot or cold stream. Therefore, heaters and coolers are run with an external source to enable heat recovery or supply. In Figure 2, all heaters and coolers use the outlet temperature specification method. As the name implies, the user defines the desired outlet temperature, and the corresponding heat duty is calculated and assigned to the associated energy stream.^17^ In the present work, the heater outlet temperatures were set according to the operational temperature of the component they feed. Meanwhile, the cooler’s outlet temperature was set to 298 K for the SOFC cathode cooler and 373 K for the afterburner cooler.

### 2.4 SOFC

The SOFC’s electrical power generation has been modeled as the product between its voltage and current (eq 5).^9,18^

4 $${Ṗ}_{\mathrm{SOFC}}=A_{c}\mathrm{jV}$$

where *A*_c_ represents the active area of the fuel cell. Current density is determined by the fuel consumption rate, *ż*, in the electrochemical reaction and the cell area, as illustrated in eq 6.

5 $$j=\frac{2Fż}{A_{c}}$$

where *F* is the Faraday constant.

The reformate stream exiting the ethanol steam reformer, comprising mainly CH_4_, H_2_, H_2_O, CO, and CO_2_, is supplied to the SOFC anode channel. At the high operating temperatures of the SOFC, CH_4_ and CO can undergo further conversion through steam reforming and water–gas shift reactions, respectively. Therefore, the fuel consumption rate, *ż*, is determined by the fuel utilization factor, *U*_f_, and the inlet molar flow rates of methane, hydrogen, and carbon monoxide, as expressed in eq 7.^9,18^

6 $$ż=U_{f}\left(4{ṅ}_{{\mathrm{CH}}_{4},\mathrm{anode}}^{\mathrm{in}}+{ṅ}_{H_{2}}^{\mathrm{in}}+{ṅ}_{\mathrm{CO}}^{\mathrm{in}}\right)$$

The hydrogen available in the anode is electrochemically oxidized according to eq 8, producing water and releasing electrons. Simultaneously, oxygen fed to the cathode is reduced to oxygen ions (eq 9), which migrate through the electrolyte to the anode side. The global electrochemical process occurring within the SOFC is summarized in eq 10. The electrons released at the anode flow through an external circuit to the cathode, thus generating direct current (DC) electricity.

7 $$a\mathrm{node}:H_{2}+O^{-2}\to H_{2}O+2e^{-}$$

8 $$c\mathrm{athode}:0.5O_{2}+2e^{-}\to O^{-2}$$

9 $$over\;all\;\mathrm{reaction}:H_{2}+0.5O_{2}\to H_{2}O$$

Operating cell voltage accounts for irreversibilities in the system, including activation (η_act_), ohmic (η_ohm_), and concentration (η_conc_) losses, as given by eq 11.

10 $$V=E_{\mathrm{OCV}}-\eta_{\mathrm{act}}-\eta_{\mathrm{ohm}}-\eta_{\mathrm{conc}}$$

The open-circuit voltage, *E*_OCV_, describes the thermodynamic voltage displayed by a reversible fuel cell. It can be obtained from the Nernst equation,^9,18,19^ which can be written as in eq 12.

11 $$E_{\mathrm{OCV}}=E^{0}-\frac{R_{u}T}{2F}\ln\left(\frac{p_{H_{2}O}}{p_{H_{2}}\cdot p_{O_{2}}^{0.5}}\right)$$

where *R*_u_ is the universal gas constant, *F* is the Faraday constant, *p*_H_2_O_ and *p*_H_2__ are the water and hydrogen partial pressures at the anode, respectively, and *p*_O_2__ is the oxygen partial pressure at the cathode. *E* ^0^ denotes the standard reversible potential, given by eq 13 and expressed as a first-order expansion about the reference temperature *T*_0_.

12 $$E^{0}=1.185+\frac{\Delta S^{{}^{\circ}}\left(T-T_{0}\right)}{2F}$$

where Δ*S*° is the standard reaction entropy.

Activation losses, η_act_, arise due to the energy required to overcome the activation barrier for an electrochemical reaction. These losses dominate at low current densities, where charge transfer kinetics limit the reaction rate. For cases where *j*_0_ ≫ *j*, i.e., small overpotential (close to equilibrium), the Butler–Volmer expression can be simplified. Therefore, the activation losses are directly proportional to the fuel cell’s temperature.

13 $$\eta_{\mathrm{act}}=\frac{R_{u}\mathrm{Tj}}{2Fj_{0}}$$

where *j*_0_ is the exchange current density, which is a function of the catalyst material and the total reaction surface area, *R*_u_ is the universal gas constant, and *T* is the temperature. Ultimately, *j*_0_ is adjusted for a better fit with experimental data.

Ohmic losses, η_ohm_, which are a consequence of Joule heating, arise due to electronic and ionic resistances in various cell components, including the anode, cathode, and electrolyte. Studies show that ohmic losses are influenced by factors such as material properties, microstructure, and thickness, with thinner anodes and cathodes reducing their contribution to overall resistance.^20^ In this study, the anode and cathode are thin compared to the electrolyte (Table 1). Thus, it can be assumed that the electrolyte plays the main role in the ohmic losses, as its ionic conductivity is much lower than the electronic conductivity of the electrodes. For instance, typical values for the ionic conductivity of electrodes are about 7 orders of magnitude higher than the conductivity of the electrolyte.^18^ It is worth mentioning that losses in the electrodes must be included for a more detailed model, especially for thicker anodes. Therefore, the ohmic voltage loss can be determined by eq 15.

14 $$\eta_{\mathrm{ohm}}=j\frac{\tau_{\mathrm{electrolyte}}}{\sigma_{\mathrm{electrolyte}}}$$

where τ_electrolyte_ and σ_electrolyte_ are the electrolyte thickness and conductivity, respectively. σ_electrolyte_ is determined by eq 16.

15 $$\sigma_{\mathrm{electrolyte}}=AT^{-1}e^{-\Delta G_{\mathrm{ac}t}/\left(R_{u}T\right)}$$

where *A* is a pre-exponential factor and Δ*G*_act_ is the activation energy for ion migration, both of which will be estimated for a better fit with experimental data.

**Table 1:** Physical Parameters of the NetxCell 7s SOFC^a^

property	anode	electrolyte	cathode
material	Ni–YSZ^b^	YSZ	LSM^c^
thickness	50 μm	150 μm	50 μm
area	28 cm^2^	100 cm^2^	28 cm^2^

a All data are available at the Fuel Cell Store^26^ website.

b Nickel and yttria-stabilized zirconia.

c Lanthanum strontium manganite.

Concentration losses, η_conc_, are caused by the depletion of reactants or accumulation of products at the catalyst layer, which hinders fuel cell performance by reducing the concentration of reactants available at the reaction site. Gas transport within the channels is dominated by convection, while gas transport within the electrodes is dominated by diffusion. Since diffusion in porous media commonly exerts the main resistance to mass transfer when compared with free convection, it will be the limiting factor.^21^ Thus, concentration losses, η_conc_, are given by eqs 17, 18, and 19.

16 $$\eta_{\mathrm{conc}}=\eta_{\mathrm{conc},a}+\eta_{\mathrm{conc},c}$$

17 $$\eta_{\mathrm{conc},a}=\frac{R_{u}T}{2F}\ln\left(\frac{P_{H_{2}O{,}_{\mathrm{TPB}}}P_{H_{2}}}{P_{H_{2}O}P_{H_{2}{,}_{\mathrm{TPB}}}}\right)$$

18 $$\eta_{\mathrm{conc},c}=\frac{R_{u}T}{4F}\ln\left(\frac{P_{O_{2}}}{P_{O_{2}{,}_{\mathrm{TPB}}}}\right)$$

where η_conc, a_ and η_conc, c_ are the concentration losses at the anode and cathode, respectively. *P*_H_2_O,_TPB__, *P*_H_2,TPB__, and *P*_O_2,TPB__ are the water, hydrogen, and oxygen partial pressures at the triple-phase boundary, while *P*_H_2_O_, *P*_H_2__, and *P*_O_2__ are the water, hydrogen, and oxygen partial pressures at the fuel cell channels. The partial pressures at the triple-phase boundary can be calculated through eqs 20, 21, and 22.^9,18,21^

19 $$P_{H_{2}O{,}_{\mathrm{TPB}}}=P_{H_{2}O}+\frac{R_{u}T\tau_{\mathrm{anode}}}{2FD_{\mathrm{eff},\mathrm{anode}}}j$$

20 $$P_{H_{2}{,}_{\mathrm{TPB}}}=P_{H_{2}}-\frac{R_{u}T\tau_{\mathrm{anode}}}{2FD_{\mathrm{eff},\mathrm{anode}}}j$$

21 $$P_{O_{2}{,}_{\mathrm{TPB}}}=P-\left(P-P_{O_{2}}\right)\exp\left(\frac{R_{u}T\tau_{\mathrm{cathode}}}{2FD_{\mathrm{eff},\mathrm{cathode}}P}j\right)$$

where τ_anode_ and τ_cathode_ are the anode and cathode thicknesses, respectively, and *D*_eff, anode_ and *D*_eff, cathode_ are the anode and cathode effective diffusion coefficients, respectively. As observed in eqs 20–22, the partial pressures at the triple-phase boundary are inversely proportional to the effective diffusion coefficients at the electrodes. Hence, electrodes that have a higher diffusion rate will exhibit a lower difference between the reactant and product concentrations at the channels and at the catalyst.

Finally, a considerable fraction of the fuel energy is converted into heat.^22^ Such heat is generated in the electrodes and transferred to the gases at the anode and cathode.^23,24^ There are three main heat generation mechanisms in an SOFC: eq 23 denotes the reversible heat generation caused by the difference between entropy consumed at the cathode and produced at the anode, eq 24 stands for the irreversible heat generation caused by the ionic transfer across the electrode–electrolyte interface, while the ohmic heating within the electrolyte due to ion conduction is given by eq 25.^25^ Consequently, the total heat output of the fuel cell is the sum of these three contributions.

22 $${\mathrm{Q̇}}_{\mathrm{rev}}=T\Delta S^{{}^{\circ}}\frac{jA_{c}}{4F}$$

23 $${\mathrm{Q̇}}_{\mathrm{irr}}=jA_{c}\eta_{\mathrm{act}}$$

24 $${\mathrm{Q̇}}_{\mathrm{ohm}}=\frac{\tau_{\mathrm{electrolyte}}A_{c}}{\sigma_{\mathrm{electrolyte}}}j^{2}$$

Finally, eq 26 is employed to calculate the required cell’s active area required to reach an α fraction of the maximal power production for a given set of thermodynamic constraints and a specific fuel flow rate. A derivation of eq 26 is given in the Supporting Information.

25 $$A_{c}=\frac{2Fż\left(\frac{\mathrm{RT}}{2Fj_{0}}+\mathrm{ASR}\right)}{\left(1-\alpha \right)E_{\mathrm{OCV}}}$$

## 3 Experimental Setup

The electrochemical performance tests of the SOFC was conducted using an 855 SOFC Test System (from Scribner Associates Inc.) equipped with a NextCell 7s Electrolyte-Supported Cell (NexTech Materials Inc.), as shown in Figure 3. Fuel cell physical parameters are listed in Table 1. To control the flow of oxidant (O_2_, 99.5% pure), fuel (H_2_, 99.95% pure), and the inert gas used for anode dilution (N_2_, 99.998% pure), needle valves were used in conjunction with a manual bubble flow meter.

**Figure 3:**
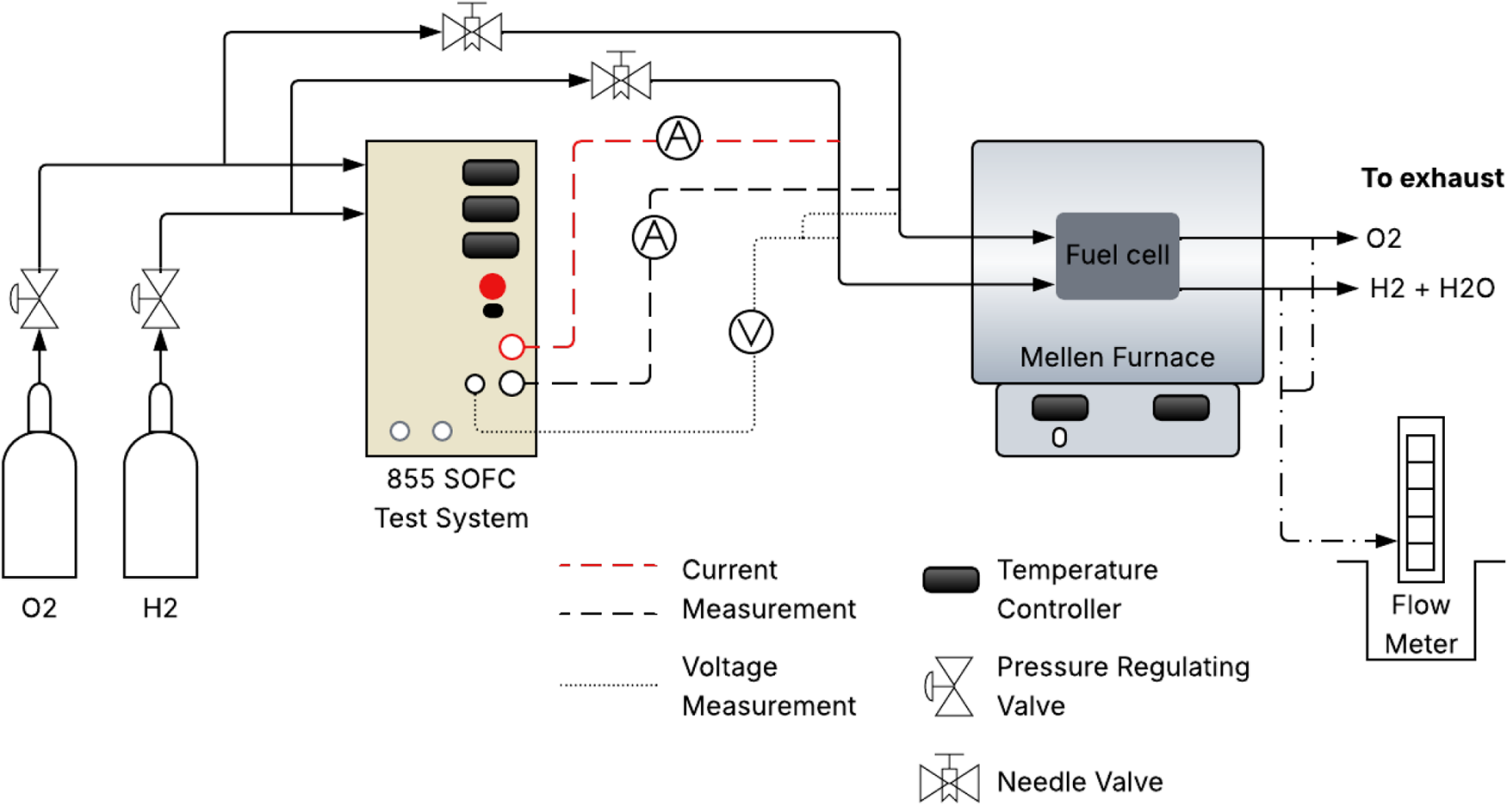
Experimental setup for the electrochemical tests and the cell assembled inside the furnace.

Before each test, the cell was conditioned by heating it up to 1123 K for 14 h (1 K/min) while maintaining a flow of O_2_ in the cathode (350 mL/min) and N_2_ in the anode (350 mL/min). Then, the nickel oxide in the anode was reduced using a mixture of 15 vol % H_2_ in N_2_. This procedure must be performed to stabilize the cell before each run test and increase the porosity of the anode.^27^ The reduction was carried out at 1123 K for 20 min, while maintaining the cathode O_2_ flow rate at 350 mL/min and the anode’s total flow rate at 260 mL/min. The process began with pure N_2_ in the anode, which was gradually incremented with H_2_. This was done to ensure that the open-circuit voltage changed in small increments. After conditioning and reduction, the flow rates were set to 260 mL/min of H_2_ and 1370 mL/min of O_2_. Once the open-circuit voltage (OCV) stabilized, testing started. The cell was tested under furnace (single-stack model from Mellen Inc., USA) temperature of 1008, 1052, and 1099 K. During the tests, initial pressures of O_2_ and H_2_ were maintained at 4.0 atm, respectively. The experimental voltage, power, and current data obtained from the 855 SOFC Test System were used to validate the SOFC model, following the procedure described in the next section.

## 4 Strategies for Parameter Estimation, Metamodel Design, and Synthesis of the Heat Exchange Network

### 4.1 SOFC Parameter Estimation against Experimental Data

In order to accurately predict SOFC performance based on experimental data, the SOFC lumped model described previously was implemented using the Python language with Cantera^28^ and Scipy^29^ libraries. The parameter estimation process began with a sensitivity analysis to identify the most influential parameters, namely, the exchange current density *j*_0_, the pre-exponential factor *A*, and the activation energy Δ*G*_act_. These parameters were then systematically varied to determine the combination that best fits the experimental data. The procedure followed the methodology proposed by Chapra and Canale,^30^ in which model parameters are optimized by minimizing the average normalized residual between predicted and measured voltages, as described in eqs 27 and 28. The model also incorporates parameters related to mass diffusion, ionic conductivity, and electrochemical kinetics, as listed in Table 3, which are consistent with values reported in the literature,^18^ unless otherwise indicated. Additional geometric parameters of the cell are listed in Table 1. Parameter optimization was carried out using SciPy’s *minimize­()* function with the *trust-constr* algorithm, which follows the trust-region approach for constrained optimization as described by Conn et al.^31^

26 $$\varepsilon_{i}=\frac{\left|V_{M,i}-V_{E,i}\right|}{V_{E,i}}$$

27 $$\mathrm{\varepsilon ̅}=\frac{1}{n}\left(\sum_{i=0}^{n}\varepsilon_{i}\right)$$

where ε_*i*_ represents the individual relative error between the operating cell voltage predicted by the model, *V*_M*, i*_, and the experimental value, *V*_E*, i*_. The term ε is the mean relative error across the *n* tests.

### 4.2 Metamodel Design

Evaluating the heat supply and demand in the reformer–SOFC system can be challenging across a wide range of input parameters, as DWSIM operates as a discrete-event simulation. To address this, a metamodel, essentially a model built from simulation results, was developed to accurately capture the response surface as a function of the input parameters while significantly reducing the number of simulation runs required. The use of metamodels to interpret simulation outcomes has been extensively discussed in the literature.^32,33^ In this study, the influence of key parameters on the reformer–SOFC system was systematically investigated by using the Response Surface Methodology (RSM) to assess their individual and interactive effects. The RSM approach was based on a Face-Centered Central Composite Design (FCCD). Analysis of variance (ANOVA) was employed to evaluate the significance of the independent variables and their interactions on the response variable, i.e., electric efficiency and heat demanded or released by the reformer and SOFC. ANOVA is a widely used technique for assessing the accuracy and significance of the experimental data. Despite the simulation’s inherent deterministic behavior, ANOVA has been successfully employed with just a single simulation run for each design point as implemented here.^34–37^ Statistica software version 13 ^38^ was used for model construction, validation, and performing the ANOVA. Table 2 lists the investigated factors along with the corresponding levels, selected based on preliminary simulation results. Additional parameters used as inputs in the DMSIM simulations are summarized in Table 3. Ethanol flow rate is set for a nominal power of 10 kW based on its LHV (lower heating value). Fuel utilization factor, *U*_f_, is defined in eq 7.

**Table 2:** Process Variables and Levels Considered for Response Surface Method (RSM) Based on Face-Centered Central Composite Design (FCCD)

	levels		
parameters	–1	0	1
oxygen-to-ethanol molar ratio	0	0.5	1.0
water-to-ethanol molar ratio	1.0	2.0	3.0
*P* [atm]	1	4.5	8
SOFC temperature [K]	1023	1073	1123
reformer temperature [K]	673	873	1073

**Table 3:** Values and Parameters Used in the SOFC Model and as an Input in the DWSIM Simulations^a^ ^b^

model	variable	value
SOFC	*A*_c_ [m^2^]	21.6 (α = 0.7)
	*A* [K/Ω· m]	59,786,504 (9.0 × 10^7^)
	Δ*G*_act_ [kJ/mol]	112.1 (100)
	*j*_0_ [A/m^2^]	10,876 (1000)
	*D*_eff, anode_ [m^2^/s]	3.66 × 10^–5^
	*D*_eff, cathode_ [m^2^/s]	1.37 × 10^–5^
DWSIM	ethanol flow rate [mol·h^–1^]	29.26
	SOFC equivalence ratio [-]	0.5
	afterburner equivalence ratio [-]	0.5
	fuel utilization factor, *U*_f_	0.85

a For *A*, Δ*G*_act_ and j_0_, initial guesses used in the regression algorithm are given in parentheses, while the optimized values. are listed.

b Note: For the SOFC model, all values were taken from the work of Saebea et al.,^18^ unless cited.

Following the FCCD, a second analysis of variance was carried out on a 3-level factorial design. For that, the following assumptions have been made: (a) the data are normally distributed within groups, (b) variance is homogeneous within each group, and (c) observations are independent from each other. Using Satistica version 13,^38^ the second-order response surface coefficients were obtained for each response variable. Since the analysis was conducted on a full factorial design, linear interactions among factors were also taken into consideration.

### 4.3 Synthesis of the Heat Exchange Network (HEN)

The synthesis of the Heat Exchange Network (HEN) was carried out using the Pinch Analysis methodology,^39^ a well-established approach for systematically identifying opportunities for heat recovery and minimizing utility consumption in chemical processes. Heat integration is restricted to hot–cold stream pairs, in accordance with the second law of thermodynamics, and a minimum approach temperature of 10 K was adopted as a design criterion.^39^

The design procedure followed the heuristics proposed by Linnhoff and Hindmarsh,^40^ which prioritize achieving stream target temperatures at each exchanger stage, thereby reducing the number of active matches in subsequent design steps and minimizing the overall network complexity. Stream data were extracted from the discrete simulation of the system using DWSIM. Streams undergoing phase change were subdivided into liquid, latent, and vapor segments, with a 1 K pseudotemperature shift applied to the latent segment to avoid divergence in specific heat. The thermal cascade was implemented in PyPinch,^41^ whose source code was adapted to calculate the energy residue according to eq 29, following the formulation in the study by Siqueira et al.^9^ In summary, the residual heat available at each temperature interval is equivalent to the sum of the heat residue from the previous temperature interval and the net heat associated with the hot and cold sources of the current interval. When the heat residue at a particular interval is negative, hot utility must be added to balance it out.

28 $$R_{k}=R_{k-1}+\sum_{i=1}^{n}{\mathrm{Q̇}}_{i,k}-\sum_{j=1}^{m}{\mathrm{Q̇}}_{j,k}$$

where *R_k_* is the energy residue in interval *k*, *Q̇*_*i,k*_ is the heat supplied by the *i*-th hot stream, and *Q̇*_*j,k*_ is the heat required by the *j*-th cold stream.

The resulting HEN was then replicated in DWSIM using a standard heat exchanger and cooler units. Hot-side outlet temperatures were calculated based on fixed cold-side outlet conditions, while utility coolers were modeled via the defined outlet temperature method. The reformer’s energy balance was enforced using the energy stream method, supported by an auxiliary Python script to comply with DWSIM stream-connection rules. Recycle blocks were included where necessary, with tolerances of 1 K for temperature and 0.1 kg/h for mass flow rate.

## 5 Results and Discussions

### 5.1 Validation of the Equilibrium Reformer Model

The Gibbs reactor model has been validated through available experimental data using the methodology proposed by Pashchenko.^42^ As illustrated in Figure 4a–c, the model reproduces the main syngas composition trends across SR and ATR conditions, while deviations arise where finite-rate kinetics and transport limitations become significant. Such discrepancies are expected, because the model enforces thermodynamic equilibrium and neglects reaction-rate constraints. Moreover, Da Silva et al.^43^ showed that ethanol reforming can exhibit metastable states departing from the equilibrium manifold under kinetically hindered regimes. The experimental data reported in the literature also suggest that the reactions proceed close to the thermodynamic equilibrium. Minor discrepancies between equilibrium predictions and experimental results were mainly observed in the hydrogen yield, typically within 15%. Given the inherent uncertainty of measurements, the equilibrium reformer model shows satisfactory agreement with the data and provides a physically consistent upper bound for conversion and heat effects at negligible computational cost, well suited to the multiscenario analysis here.

**Figure 4:**
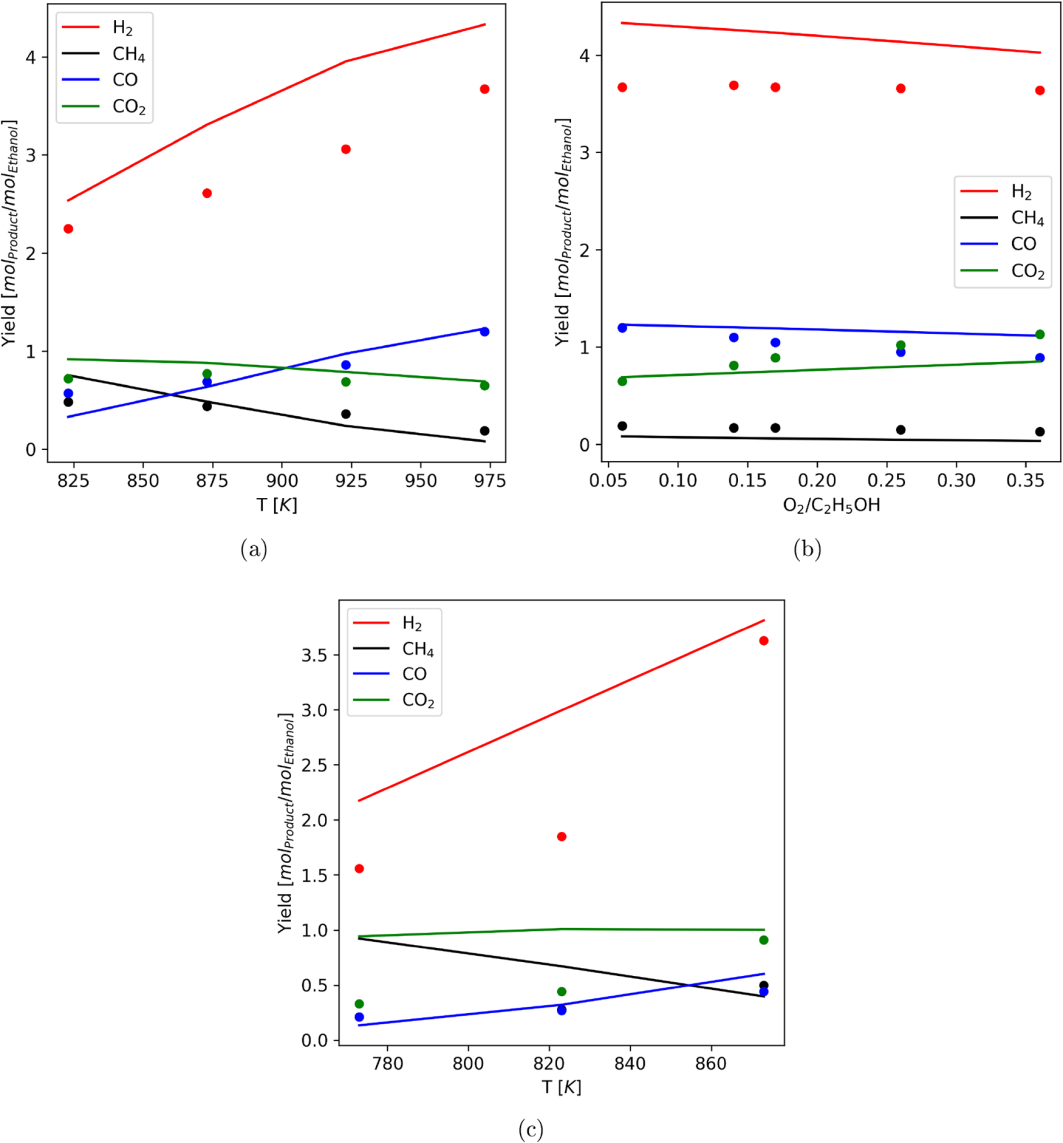
Comparison of the Gibbs reactor model predictions against: (a) autothermal reforming over a NiCeAl catalyst under H_2_O/C_2_H_5_OH = 3 and O_2_/C_2_H_5_OH = 0.06,^44^ (b) autothermal reforming over a NiCeAl catalyst under H_2_O/C_2_H_5_OH = 3 and *T* = 973 K,^44^ and (c) autothermal reforming over a Rh/C*e*_1/2_Z*r*_1/2_O_2_ catalyst under H_2_O/C_2_H_5_OH = 4 and O_2_/C_2_H_5_OH = 0.^45^

### 5.2 SOFC Performance and Experimental Validation of the Lumped Model

Figure 5 provides a comparison between the experimental data and model predictions of the fuel cell. Overall, the experimental and predicted power output and voltage curves exhibit similar trends across the three temperature conditions (1008, 1052, and 1099 K), with a curve shape consistent with those reported in the literature.^46–50^ As expected, the model diverges to predict power and voltage at higher temperatures and at higher current densities, indicating that the simplified lumped model does not fully capture the associated losses under these conditions. Refining activation overpotential with a more realistic *j*_0_ will be necessary for a higher accuracy, e.g, using a nonlinearized approach to the Butler–Volmer equation as elaborated in detail by Siqueira et al.^9^ Even though the trends suggest that additional refinements to the model might improve accuracy, the average error predicted by eq 28 is lower than 2% (Table 4).

**Figure 5:**
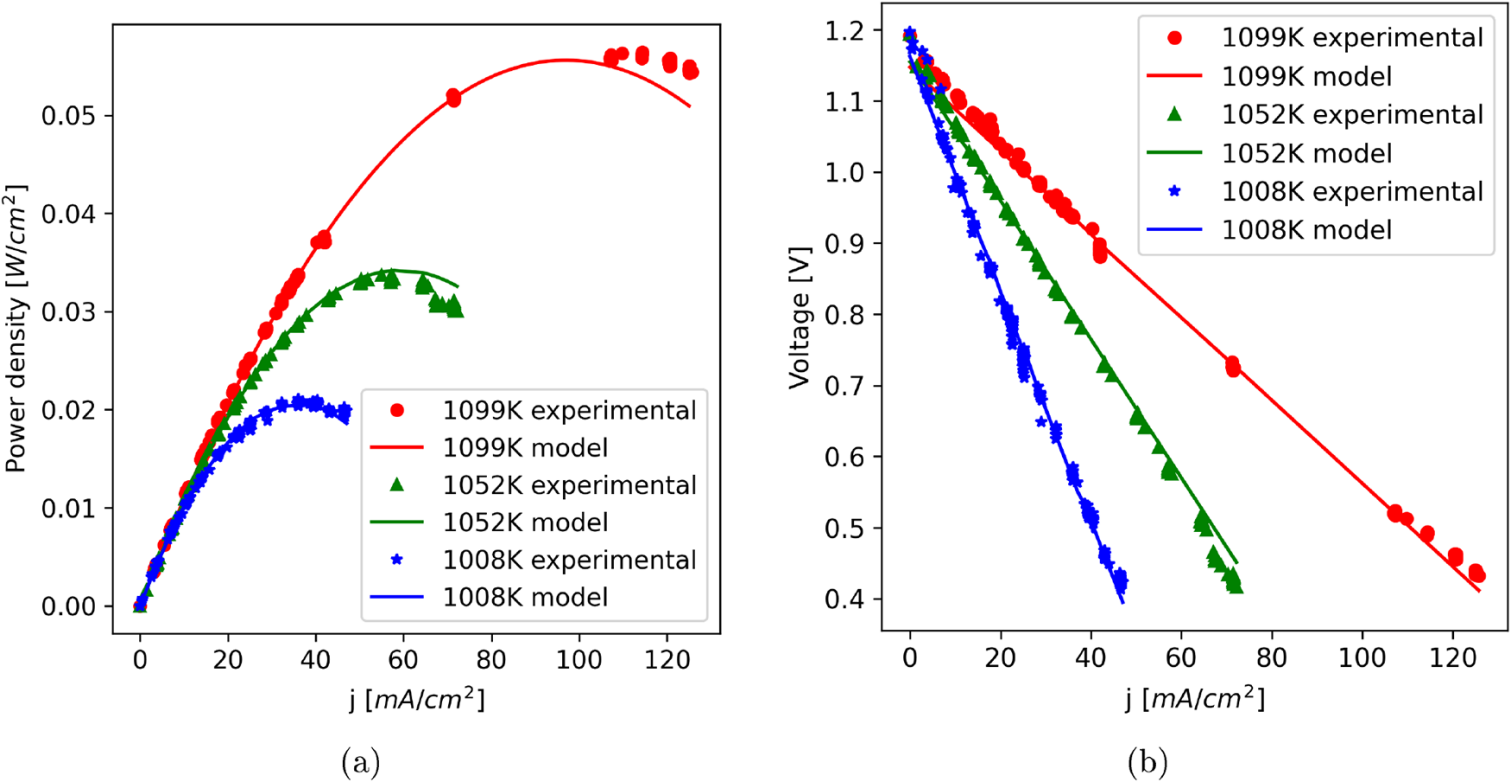
Comparison between experimental data and lumped model predictions: (a) power curve and (b) polarization curve.

**Table 4:** Optimized Parameters and Validation Metrics for SOFC Datasets from the Literature

	this work	Li et al.^47^	Neri et al.^49^	Hung and Shy^48^	Leah et al.^46^	Tikiz et al.^50^
*A* [K/Ω · m]	5,97,865 × 10^7^	5,97,864 × 10^7^	5,97,865 × 10^7^	5,97,865 × 10^7^	5,97,865 × 10^7^	5,97,865 × 10^7^
Δ*G*_act_ [kJ/mol]	112.1	94.4	107.8	116.5	86.3	79.2
*j*_0_ [A/m^2^]	10876	18417	945	909	549	1623
average error [%]	1.56	1.26	0.98	3.85	9.45	18.66
maximum error [%]	9.89	2.57	4.79	12.69	29.86	124.07

The model was also evaluated with experimental data from the literature covering a wide range of pressures and temperatures.^46–50^ Leah et al.^46^ conducted experiments on a metal-supported IT–SOFC at 1.2 atm and 823, 843, and 873 K. Li et al.^47^ used a single electrolyte-supported cell at a pressure of 1 atm and temperatures of 1019, 1068, 1116, and 1165 K fed with H_2_, H_2_O, and N_2_. Neri et al.^49^ tested an anode-supported SOFC at 1 atm and temperatures between 823 and 973 K, with 7% humidified H_2_ and dry air. Similarly, Tikiz et al.^50^ tested an anode-supported SOFC between 973 and 1073 K at pressures of 3, 4, and 5 atm using H_2_ and O_2_. Finally, Hung and Shy^48^ used H_2_ and N_2_ as fuels for an anode-supported SOFC at temperatures between 1023 and 1123 K and pressures of 1, 3, and 5 atm. For each case, the parameter estimation procedure described in Section 4.1 was applied. The resulting best-fit parameters are presented in Table 4, and parity plots comparing the modeled and experimental data are shown in Figure 6.

**Figure 6:**
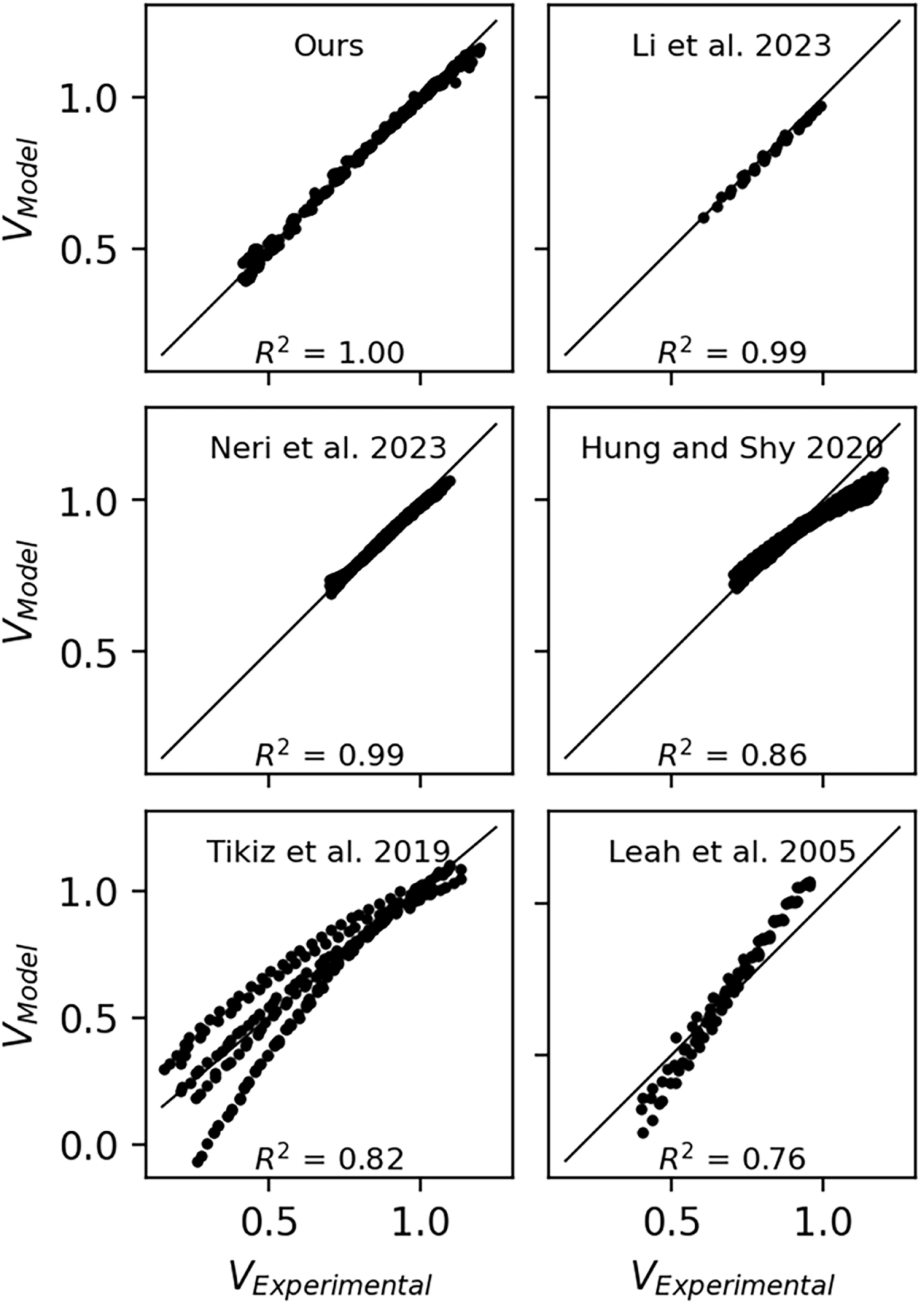
Parity plots comparing predicted and experimental data.

The model achieved the best agreement with the results of Li et al.^47^ and Neri et al.,^49^ with errors primarily near open-circuit conditions, where thermodynamic voltage and activation overpotentials are dominant. In these cases, the model overestimated voltage losses, likely due to simplified assumptions about local partial pressures. In the case of Neri et al.,^49^ the use of an anode-supported configuration may introduce minor deviations associated with electrode thickness and mass transport limitations. While Hung and Shy^48^ also employed anode-supported cells, and their results showed larger deviations, possibly attributed to fuel dilution effects. In contrast, the model underestimated losses in the study by Leah et al.,^46^ which can be attributed to the lower operating temperatures and use of a metal-supported architecture, both of which affect hydrogen and water vapor partial pressures. The largest discrepancies were observed in the data set of Tikiz et al.,^50^ where the model captured low-current behavior accurately but failed to reproduce performance at higher current densities, revealing limitations in representing activation and concentration losses. This deviation is primarily attributed to the simplifications adopted in the lumped model. At high current densities, the linearized Butler–Volmer formulation underestimates activation overpotentials, while mass transport and temperature gradients within the electrodes are not explicitly captured. Consequently, the model slightly overpredicts the performance under extreme operating conditions, although its accuracy remains sufficient for system-level steady-state analysis and heat integration studies.

Despite these limitations, the lumped model demonstrated good overall agreement across a variety of SOFC designs and operating conditions, with average errors below 2% in most cases. Its computational efficiency and predictive accuracy make it suitable for integration into flowsheet simulations using DWSIM, enabling the exploration of system-level configurations and thermal integration strategies.

### 5.3 Metamodel Development and Results

Before the full analysis of the system parameters was carried out, the three main reforming reactions were evaluated separately at 1 atm, with the reformer and SOFC temperatures set to 873 and 1073 K, respectively. The results presented on Table 5 have been obtained by maximizing the electrical efficiency within the constraints of a single reforming type. Under such optimized conditions, the three reforming routes yield essentially the same electrical efficiency because operating shifts that boost power are offset by proportional increases in heater energy demand.

**Table 5:** Ethanol Steam Reforming, Partial Oxidation, and Autothermal Reforming Reactions: Key Performance Indicators under Optimized Conditions

reaction	H_2_O/C_2_H_5_OH	O_2_/C_2_H_5_OH	*Ṗ*_SOFC_ [kW]	η_elec_ [-]	*Ṗ*_heaters_ [kW]
steam reforming	1.11	0	5.32	0.364	4.63
partial oxidation	0	0.38	4.79	0.363	3.17
autothermal reforming	0.94	0.19	5.14	0.362	4.17

#### 5.3.1 ANOVA with 5-Factor Central Composite Design

The DWSIM flowsheet simulation was analyzed using a central composite design of experiments, considering five input variables, as listed in Table 2. The system electrical efficiency was selected as the primary response variable. This simulation framework also enabled the evaluation of the heat load distribution across heat balances for the components under the defined operating conditions. The complete set of discrete simulation results is available in the Supporting Information (Table S1). In terms of heat demand, the primary consumers were the heaters associated with the SOFC air supply and the water–ethanol mixture that was fed to the reformer, which together accounted for most of the system heat input. On the heat recovery side, the dominant contributors were the excess heat in the SOFC cathode exhaust and the heat generated in afterburner. Across all evaluated conditions, the system electrical efficiency remained below 44%. This outcome highlights the significant efficiency penalty associated with the lack of heat integration between the exhaust and reactant streams. Therefore, thermal coupling strategies between these elements are essential to improve overall system performance.

To evaluate the influence of each factor on electrical efficiency, an analysis of variance (ANOVA) was performed by using Statistica software, assuming a 95% confidence level. The resulting main effects model achieved an *R* ^2^ value of 0.84774, indicating a good fit and an adequate explanation of the variability in the response. The complete ANOVA results are presented in Table S2 in the Supporting Information. The analysis confirmed that the most significant variables were the reformer temperature (*T*_REF_) and the SOFC temperature (*T*_SOFC_). Since the reformer is assumed to be in equilibrium, the hydrogen mole fraction in the products is highly impacted by the operational temperature, indicating that the reactant proportions and pressure play a lesser (albeit important) role on its production. On the SOFC side, the electrolyte conductivity is inversely proportional to the temperature, implying that ion mobility decreases at lower temperatures. Similarly, activation and concentration losses also increase with temperature further degrading the performance. Nevertheless, the open-circuit voltage also decreases at lower temperatures, which means that SOFC performance will be affected by the temperature regardless of the power output. Finally, higher temperatures increase the energy demand to heat up the reactants, limiting the efficiency that can be achieved. All of these factors contribute to make the temperature the most relevant parameter for the system’s overall performance.

#### 5.3.2 ANOVA with 2-Factor Full Factorial Design

To enable a comprehensive and systematic comparison of the effects of the main ethanol reforming pathways, e.g., steam reforming, partial oxidation, and autothermal reforming, on system heat integration, a simplified design of the experiments was adopted. This simplification involved fixing *T*_SOFC_, *T*_REF_, and the system pressure *p*, while allowing the variation of the oxygen-to-ethanol and water-to-ethanol molar ratios. The complete set of results from the discrete simulation involving just two factors is available in the Supporting Information (Table S3). Based on the previous studies by Liu et al.,^2^ Hu et al.,^51^ Siqueira et al.,^9^ and O’Hayre et al.,^21^ high SOFC operating temperatures are essential for maximizing the power output. Accordingly, *T*_SOFC_ was fixed at 1073 K, in line with the upper limits reported in those works. Similarly, elevated reformer temperatures are known to enhance the ethanol conversion efficiency. As shown by Mosayebi^52^ and Chen et al.,^53^ optimal temperatures for ethanol reforming range from 723 to 873 K for steam reforming and 573–873 K for partial oxidation and autothermal reforming. Therefore, *T*_REF_ was fixed at 873 K. Since the influence of temperature is well-established in the literature, fixing these parameters allowed for a more targeted investigation of the remaining variables. To further streamline the experimental matrix, the system pressure was fixed at 1 atm. This refined analysis included multiple response variables: electrical efficiency, SOFC heat load, reformer heat load, heat demand from the heaters, and heat recovery from the cathode and anode. A three-level full factorial design was applied, and the analysis of variance (ANOVA) was performed assuming a 95% confidence level. The associated p-values, which accounts for linear, quadratic, and interaction effects, are presented in Table 6.

**Table 6:** *P*-values from the 2-Factor Full Factorial Design

factor^a^	η_elec_	*Q̇*_SOFC_	*Q̇*_Reformer_	*Q̇*_Heaters_	*Q̇*_Cathode_	*Q̇*_Anode_
*X*_1_	0.000011	0.211177	0.000004	0.000000	0.000002	0.018337
*X*_1_^2^	0.180057	0.418772	0.017281	0.007700	0.003021	0.439118
*X*_2_	0.000001	0.000075	0.000000	0.000002	0.000018	0.216868
*X*_2_^2^	0.000911	0.658897	0.000073	0.000205	0.000066	0.543411
*X*_1_ *X*_2_	0.000533	0.144454	0.000028	0.000061	0.000020	0.201503

a *X*_1_ and *X*_2_ stand for water-to-ethanol and oxygen-to-ethanol molar ratios, respectively.

The ANOVA results presented in Table 6 reveal distinct sensitivities of each response variable to the independent factors *X*_1_ (water-to-ethanol) and *X*_2_ (oxygen-to-ethanol). Electrical efficiency (η_elec_) was influenced by all terms except the quadratic component of *X*_1_, indicating a strong linear dependency on both molar ratios as well as with the interaction between them. For the SOFC heat load (*Q̇*_SOFC_), only the linear term of *X*_2_ was statistically significant. The type of reform reaction is controlled mainly by the oxygen content, which suggests that the fuel cell’s thermal output linear dependence in regard to the oxygen is closely linked to the amount of hydrogen available in the syngas. In the case of the reformer heat load (*Q̇*_Reformer_), all terms, including quadratic and interaction effects, were highly significant, indicating complex, nonlinear interactions between the two input variables. A similar trend was observed for the heater demand (*Q̇*_Heaters_) and cathode-side heat recovery (*Q̇*_Cathode_), both of which showed full statistical significance to all factors, emphasizing the strong coupling between fuel composition and thermal balance in these subsystems. Finally, the anode heat recovery (*Q̇*_Anode_) was significantly affected only by the linear component of *X*_1_, suggesting that it is predominantly influenced by the water-to-ethanol ratio, with limited dependence on the oxygen content or higher-order interactions.

The resulting response surfaces follow a second-order polynomial form, as expressed by eq 30. Under the fixed operating conditions (1 atm, reformer temperature of 873 K, and SOFC temperature of 1073 K), these surfaces were well represented by the regression models listed in Table 7. As shown, all regression models exhibit high coefficients of determination (*R* ^2^), confirming the fit between the predicted and simulated data. This suggests that the second-order polynomial structure is highly appropriate for capturing the nonlinear dependencies of these variables on the reactant molar ratios.

29 $$Y=a_{0}+a_{1}X_{1}+a_{2}X_{1}^{2}+a_{3}X_{2}+a_{4}X_{2}^{2}+a_{5}X_{1}X_{2}$$

**Table 7:** Regression Coefficients of the Response Variables

variable	*a*_0_	*a*_1_	*a*_2_	*a*_3_	*a*_4_	*a*_5_	*R* ^2^
η_elec_ [-]	0.391085	–0.032155	0.001085	–0.079732	–0.033253	0.014087	0.99983
*Q̇*_SOFC_ [kW]	–3.09541	–0.27209	0.04210	1.43885	–0.08792	0.12499	0.99687
*Q̇*_Reformer_ [kW]	0.23655	0.32395	–0.01602	–2.34977	–0.41670	–0.20293	0.99999
*Q̇*_Heaters_ [kW]	2.461464	1.543885	–0.065038	2.999004	–0.894333	–0.475060	0.99993
*Q̇*_Cathode_ [kW]	–0.92786	–0.91587	0.06036	–2.18426	0.87474	0.46038	0.99984
*Q̇*_Anode_ [kW]	–3.26923	0.13260	–0.05885	0.36671	–0.18079	–0.15250	0.90426
*Q̇*_Net_ [kW]	–4.59448	0.81247	–0.03745	0.27054	–0.70501	–0.24512	0.99902

Figure 7 presents the response surfaces for electrical efficiency (η_elec_), SOFC heat load (*Q̇*_SOFC_), and reformer heat load (*Q̇*_Reformer_) as functions of the water-to-ethanol (*X*_1_) and oxygen-to-ethanol (*X*_2_) molar ratios. Since the fuel flow rate was kept constant, electrical efficiency is directly correlated to the SOFC electrical power output. As shown in Figure 7a, the efficiency increases under fuel-rich conditions, particularly at lower *X*_2_ values. The maximum efficiency of 36% was achieved at a water-to-ethanol ratio of 1.0 and an oxygen-to-ethanol ratio near zero, indicating favorable operating conditions for steam reforming with minimal oxygen addition. Although the power generation increases with higher water-to-ethanol ratios (Figure 7b), the energy demanded to heat up the reactants grows significantly faster. For this reason, lower water-to-ethanol ratios lead to higher efficiency. The SOFC heat load surface (Figure 7c) mirrors this behavior, with heat output increasing as less oxygen is introduced. All other things being equal, less oxygen for the reformer increases the hydrogen content of the syngas. Hence, more fuel is consumed at the SOFC, generating more power and heat. This heat represents a valuable internal energy stream that can be redirected to support other thermal demands in the system. Conversely, the reformer heat load (Figure 7d) is highly sensitive to the oxygen content. At low *X*_2_ values, the reformer operates in an endothermic regime, requiring significant external heat input, particularly under steam reforming conditions. As the oxygen content increases, partial oxidation reactions reduce the reformer net heat demand, and at oxygen-to-ethanol ratios above approximately 0.4, the reformer becomes exothermic. The precise location of the thermoneutral (autothermal) point depends on the combined effect of both *X*_1_ and *X*_2_, reflecting the complex interplay between steam dilution and oxidation.

**Figure 7:**
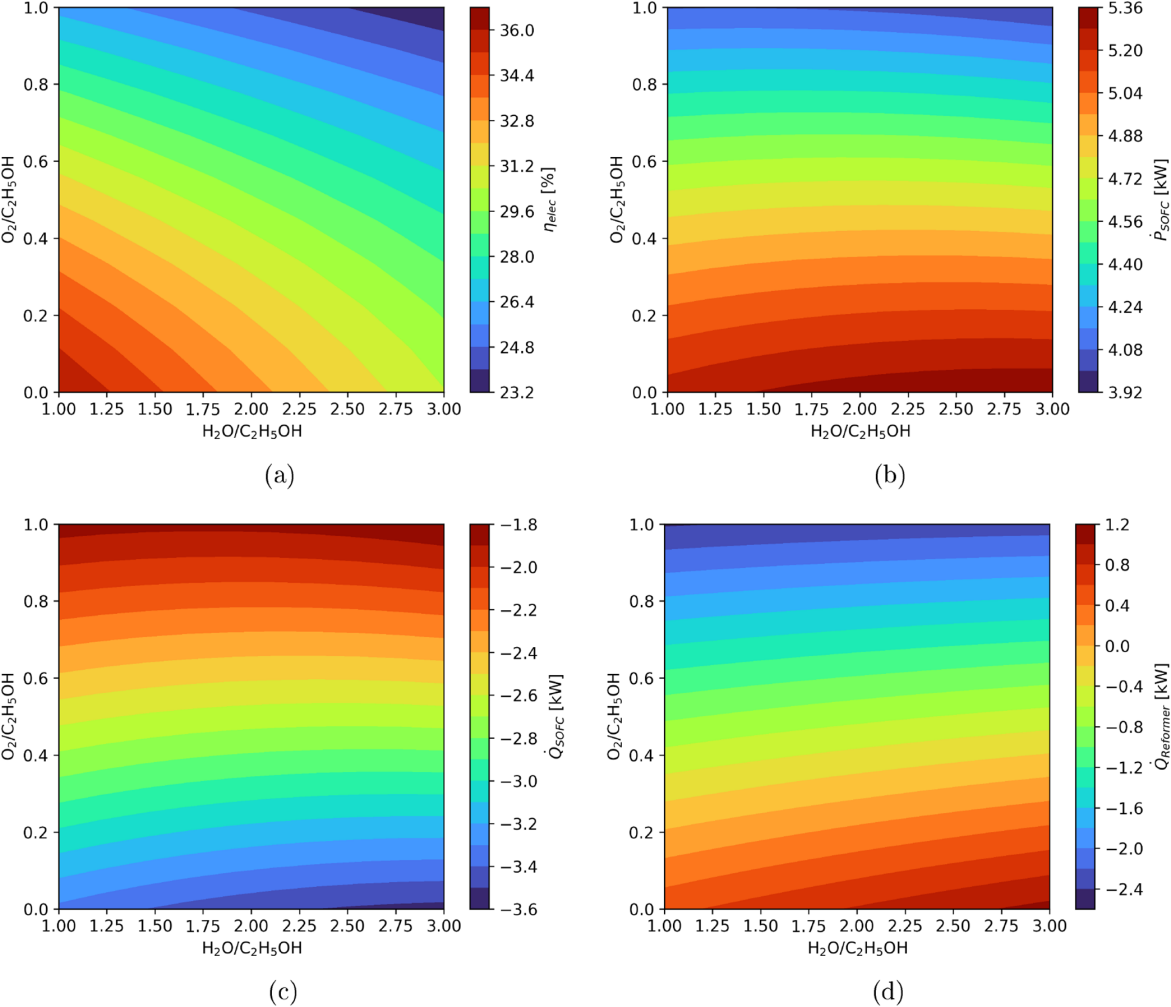
Response surfaces for (a) electrical efficiency (η_elec_), (b) SOFC power output (*Ṗ*_SOFC_), (c) SOFC heat load (*Q̇*_SOFC_), and (d) reformer heat load (*Q̇*_Reformer_).

These observations first reveal opportunities for heat integration grounded in the First Law of Thermodynamics. The surplus heat released by the SOFC, especially under fuel-rich, low *X*_2_ conditions, can be used to supply the thermal energy required for reforming, thus reducing or even eliminating the need for external heating sources. Moreover, by operating near the autothermal reforming region, where the reformer net heat load approaches zero, the system can achieve thermal self-sufficiency with minimal external intervention. The temperature gradient between the SOFC (operating at 1073 K) and the reformer (873 K) further facilitates an effective heat exchange. From a system integration standpoint, these findings highlight the potential for cascading thermal energy from the SOFC to the reformer and even to auxiliary components such as feed preheaters, thereby enhancing overall energy utilization.

Although the optimal conditions are promising in terms of heat integration, they might lead to system longevity issues due to catalyst deactivation. As reviewed by Chen et al.,^53^ carbon deposition is a well-documented issue in ethanol reforming, particularly under low water–ethanol ratios and in the absence of oxygen. Such carbon deposits block the catalyst’s active sites, reducing the production of hydrogen. As a consequence, the fuel cell would produce less power and heat. Nevertheless, such conditions might enable a higher fraction of unused fuel to pass through the SOFC, which could cause a shift in the equivalence ratio of the afterburner.

### 5.4 System-Level Heat Integration Insights

Figure 8 presents a comprehensive thermal analysis of the system, encompassing the total heat demand from heaters (Figure 8a), the recoverable heat from the SOFC cathode (Figure 8b) and anode (Figure 8a), and the system net heat production (Figure 8d). Figure 8a quantifies the heater duty across the design space and shows that heater loads can reach up to 74% of the total fuel energy input. This underscores the critical importance of minimizing external heating requirements to enhance the electrical efficiency. The recoverable heat shown in Figure 8b,c indicates that a significant portion of this demand could be internally supplied, particularly if the system includes integrated heat exchange units leveraging the high exhaust temperatures of the SOFC (1073 K) to preheat feed streams entering at lower reformer temperatures (873 K).

**Figure 8:**
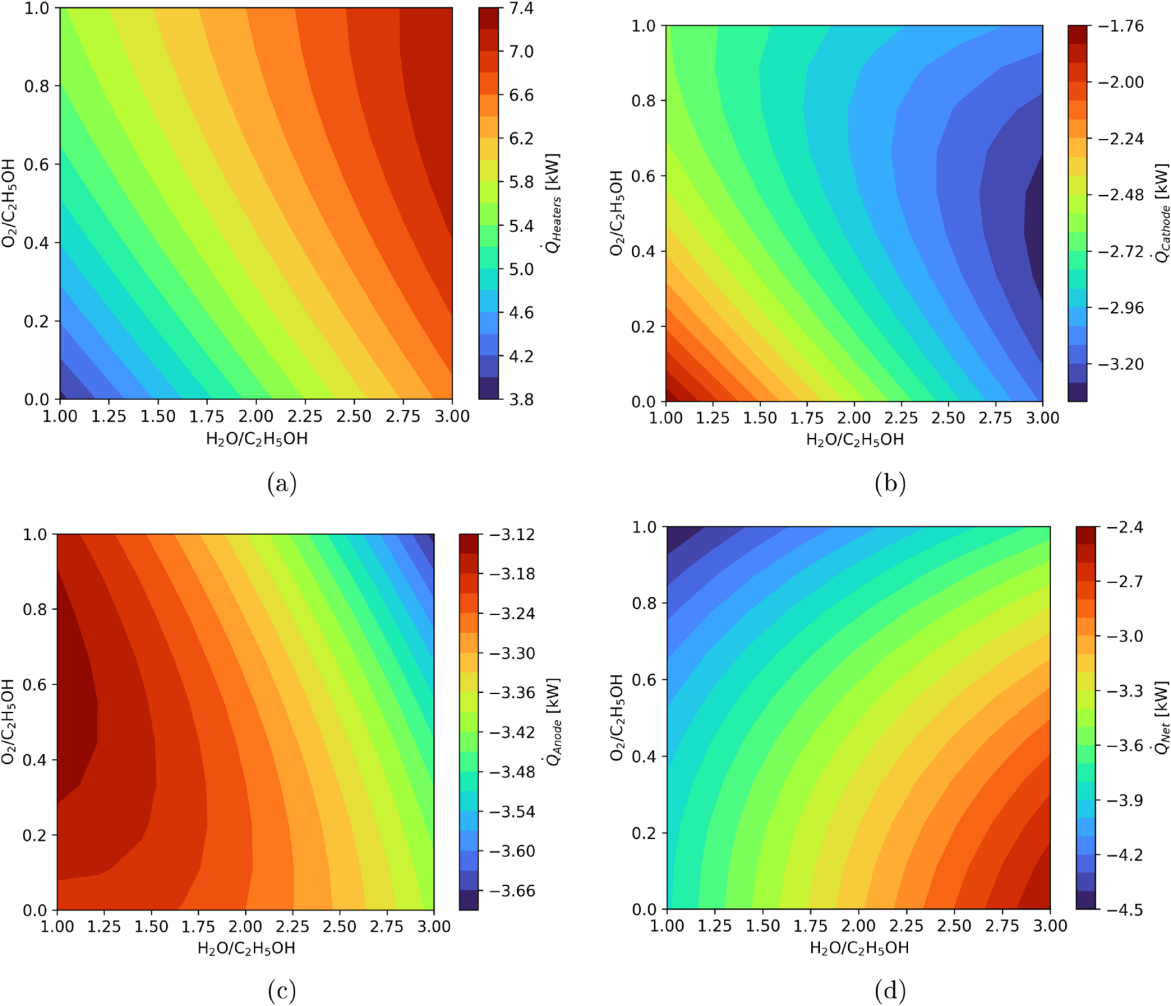
Heat distribution across system components: (a) heaters demand (*Q̇*_Heaters_), (b) cathode generation (*Q̇*_Cathode_), (c) anode generation (*Q̇*_Anode_), and (d) net system heat (*Q̇*_Net_).

The net heat production shown in Figure 8d further highlights a key thermodynamic trade-off: as the SOFC heat output increases, typically under fuel-rich conditions or a low oxygen-to-ethanol ratio, the net heat available for recovery decreases. This occurs because the incremental heat generation is outpaced by the growing heat demand from increased water and air feeds. Meanwhile, the water-to-ethanol ratio modulates the heat demand side of the balance. Higher water content elevates the reformer heating needs due to vaporization and preheating but also increases the recoverable heat from the SOFC exhaust due to the higher hydrogen contents in reformed gas, as shown in Figure 8b,8c. This suggests that the water-to-ethanol ratio can be employed as a control variable to fine-tune heat integration, offering a strategy to match available thermal energy with internal process demands. Nevertheless, the system remains net exothermic over the entire range, confirming a thermodynamic window where heat integration is not only possible but advantageous.

From a design standpoint, these results emphasize several integration opportunities. First, direct thermal coupling between the SOFC and reformer through recuperative heat exchange can reduce or eliminate the need for external heaters. Second, the excess heat recovered from the SOFC’s cathode and anode exhausts can be redirected to support ancillary loads, such as water vaporization or feed preheating. Third, operation near autothermal reforming conditions minimizes the internal heat redistribution requirements, potentially simplifying control strategies and hardware.

Although the present analysis is based on a simplified energy balance and does not yet incorporate second-law (exergy) considerations, the results strongly support the technical feasibility of a self-sustaining, thermally integrated SOFC–reformer system. Future work should expand upon these findings by designing explicit heat integration schemes, e.g., via pinch analysis or heat exchanger network synthesis, assessing their dynamic behavior under transient conditions and quantifying their impacts on system efficiency and reliability.

### 5.5 Synthesis of the Heat Exchange Network (HEN) and Evaluation of Efficiency Improvement

In principle, the temperatures and heat capacities of process streams vary with the operating conditions, which implies that the synthesis of a Heat Exchange Network (HEN) would yield different configurations across the design space. Therefore, to test the potential for heat integration, a representative HEN was designed for a single operating point corresponding to the highest electrical efficiency: water-to-ethanol ratio of 1.0, oxygen-to-ethanol ratio of 0.0, pressure of 1 atm, reformer temperature of 873 K, and SOFC temperature of 1073 K. The corresponding stream data extracted from the flowsheet simulations are reported in Table S4 of the Supporting Information. This data set was processed using PyPinch,^41^ applying a minimum temperature difference of 10 K, which produced the heat cascade shown in Table S5.

Following the pinch design heuristics, an initial HEN topology was developed. As shown in Figure 9, the proposed configuration prioritizes the recovery of heat from the SOFC cathode exhaust and the afterburner to preheat the cold reactant streams. It must be noted that C1, C2, and C3 (Table S4) correspond to the segments of the same material stream (the water/ethanol mixture), while H1, H2, and H3 (also Table S4) represent the subdivisions of the SOFC coolant. These subdivisions can be combined into single exchangers in the final network, reducing its apparent complexity. By contrast, C5 and C6 cannot be merged, since they stem from distinct modeling approaches: C5 from the isothermal reactor’s energy balance and C6 from a conventional material stream. Consequently, these two exchangers were retained as independent units in the final design.

**Figure 9:**
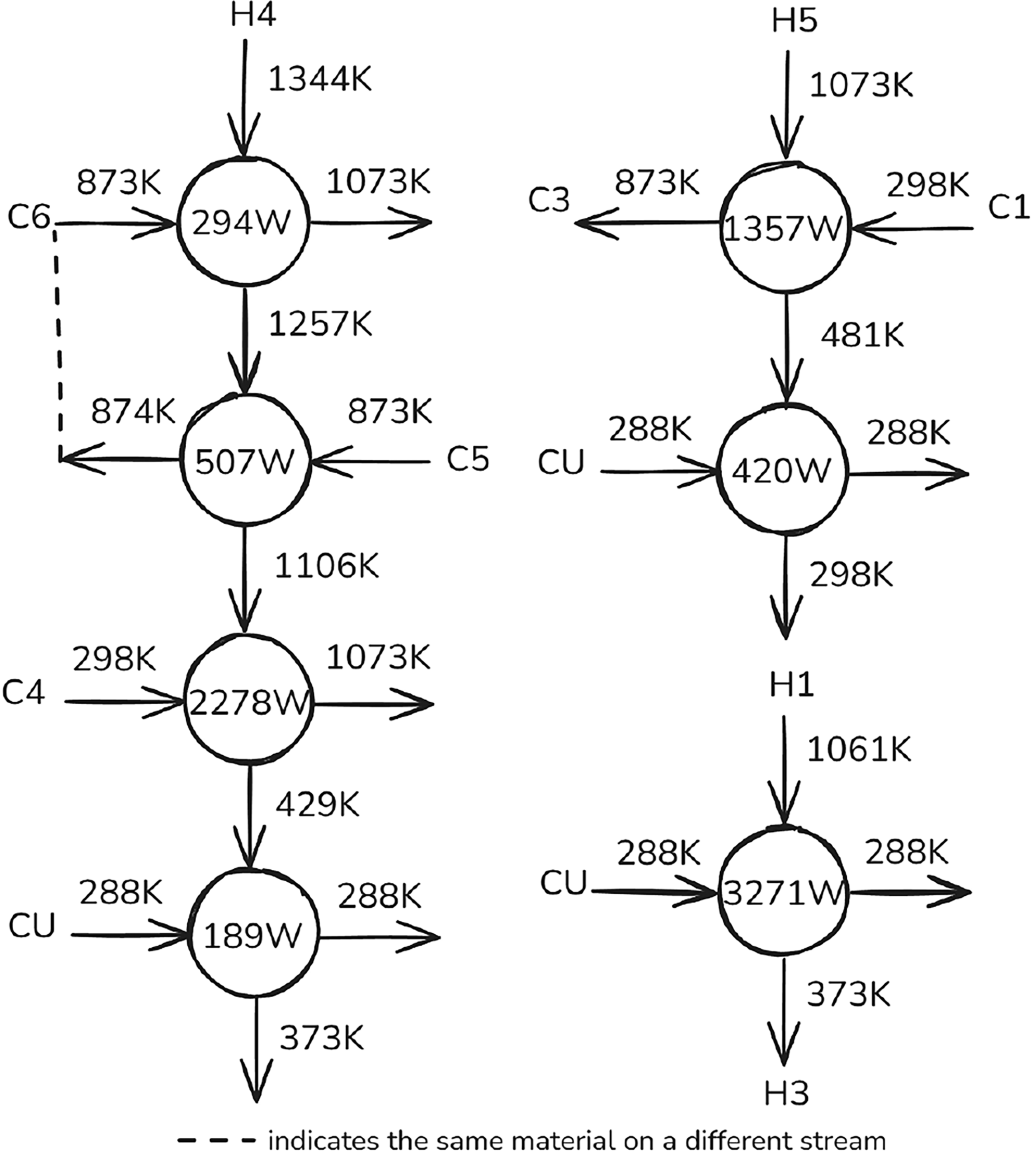
Proposed reduced topology for the Heat Exchange Network.

The proposed HEN was implemented in DWSIM, resulting in the flowchart shown in Figure 10. Comparison of the simulation results with the pinch design parameters (Table S6) indicates that the pinch method slightly overestimates the temperature drop in hot streams. However, the deviation did not exceed 2% for temperature predictions, while the calculated energy exchange between streams was within ±0.5% of the simulation values. Importantly, the design ensured that the heating requirements of all reactant streams were fully met without the need for hot utilities. It is important to rise that the proposed HEN is synthesized at the design point and each heat exchanger is sized to meet specified outlet temperatures. When operating conditions depart from this point, certain matches may violate Δ*T*_min_ or experience duty-sign reversal, e.g., when moving from SR to POX, rendering the original network infeasible and causing flowsheet nonconvergence. A comprehensive off-design treatment thus requires resynthesis of the HEN for each operating point.

**Figure 10:**
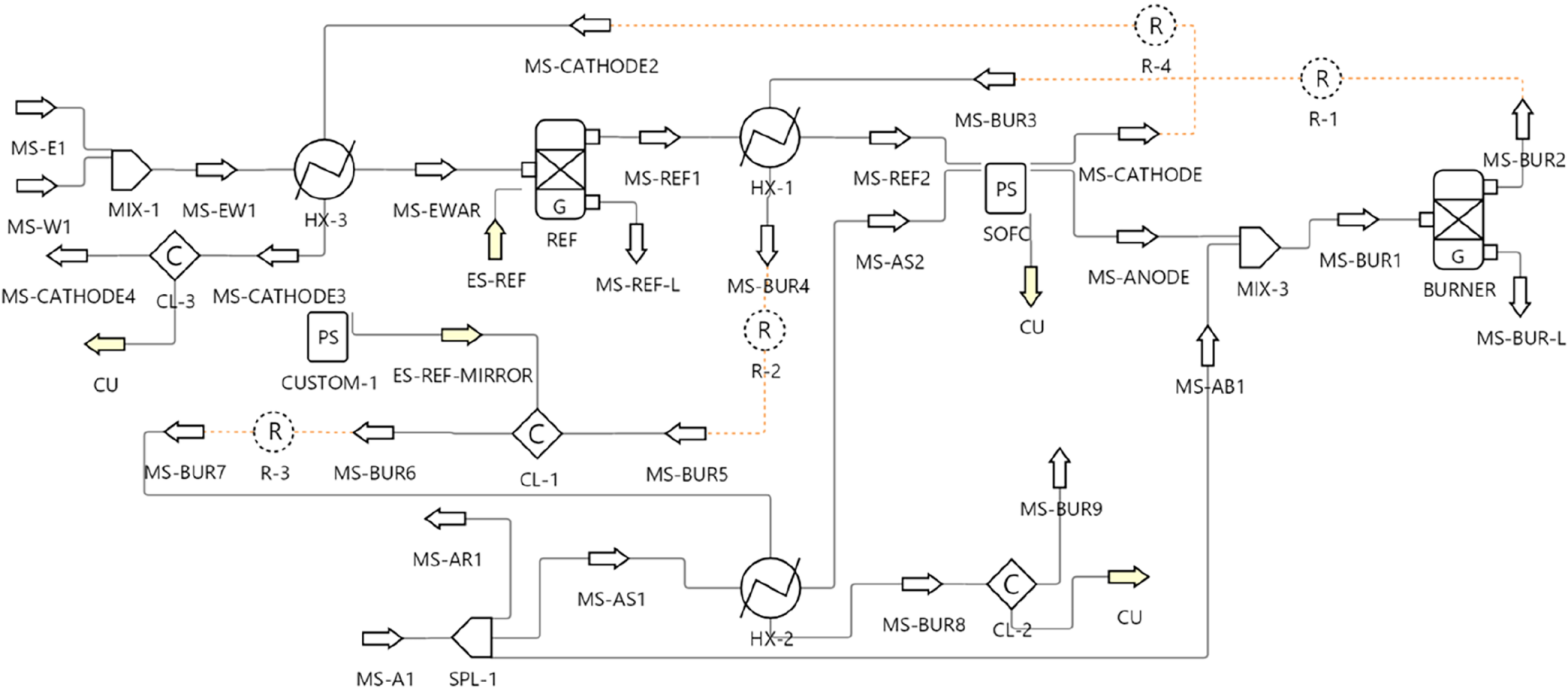
Flowsheet simulation of the Heat Exchange Network (HEN) integrated into the SOFC–reformer system.

Overall, the results show that the proposed HEN can effectively balance the heat supply and demand in the system. When compared with the baseline nonintegrated configuration (without internal heat recovery), the electrical efficiency increases from 36 to 52%. This demonstrates the significant potential of systematic heat integration to enhance the performance of SOFC–ethanol reformer systems while reducing reliance on external utilities and reinforcing the role of pinch-based design as a reliable tool for integrated energy management.

## 6 Conclusions

This study developed a unified modeling framework to evaluate the thermal integration potential between solid oxide fuel cells (SOFCs) and ethanol reformers operating under steam reforming, partial oxidation, and autothermal reforming pathways. A validated lumped SOFC model was implemented in a flowsheet simulation environment and applied to a broad design space, enabling a comprehensive assessment of the system behavior and heat integration opportunities. To support the interpretation of simulation results, an analysis of variance (ANOVA) was conducted to construct a metamodel, which provided insights into the influence of key process variables and facilitated the identification of favorable conditions for thermal integration.

System performance was found to be highly sensitive to the operating temperatures of both the reformer and the SOFC, with optimal conditions identified at 873 and 1073 K, respectively. Under these settings, response surface models achieved *R* ^2^ values above 0.99 for most outputs, confirming that second-order regressions accurately captured the system behavior. The maximum electrical efficiency of 36% occurred at low oxygen-to-ethanol ratios and a water-to-ethanol ratio near 1.0, corresponding to the minimum overall heat demand and revealing the strong thermal coupling between the reformer and the SOFC. From a first-law thermodynamic perspective, the SOFC consistently acted as a net heat source, while the heat demand in the reformer could be balanced through internal recovery. This insight was validated through the synthesis of a Heat Exchanger Network (HEN) based on the Pinch method, which, when implemented in DWSIM, achieved full internal heat recovery and enhanced electrical efficiency from 36 to 52%, effectively eliminating the need for an external heat supply under steady-state operation.

Although the present analysis focused on steady-state conditions, dynamic operation aspects such as start-up, shutdown, and load-following are expected to influence the transient thermal balance and, consequently, the performance of the integrated system. During start-up, the SOFC and reformer components reach their operating temperatures at different rates, potentially limiting the effectiveness of the proposed HEN until thermal equilibrium is achieved. Similarly, load variations may shift the heat integration point, requiring adaptive control strategies to maintain the thermal stability. Practical implementation also faces material and control constraints, including thermal stress, degradation of catalysts, and the need for precise flow management. In addition, extending the present first-law thermodynamic framework to include a second-law (exergy) analysis would allow for the identification of irreversibilities and improvement potentials, thereby complementing the heat integration results and further strengthening the thermodynamic evaluation of the system.

In summary, this work demonstrates that SOFC–reformer configurations fueled by ethanol can achieve thermally self-sustained operation under steady-state conditions when properly optimized. Beyond its technical findings, this work advances a generalized modeling framework that bridges the process design and thermodynamic analysis, providing a transferable methodology for evaluating other fuel-flexible energy systems. It also underscores the potential of ethanol as a renewable hydrogen carrier for SOFC-based power generation. These contributions collectively highlight the broader significance of thermally integrated SOFC–ethanol reformer systems in the development of compact, high-efficiency, and low-emission energy technologies.

## Supplementary Material



## Notes

Simulation results for five- and two-factor DoE (PDF)

## List of Symbols

*A*: pre-exponential factor [K/(Ω · m)]
*A*_c_: active area of SOFC [m^2^]
*ASR*: area-specific resistance [Ω · m^2^]
α: ratio between the finite-area and infinite-area fuel cell power output [−]
*c*_p_: specific heat capacity [J/(kg · K)]
*D*_eff, anode_: effective diffusion coefficient in the anode [m^2^/s]
*D*_eff, cathode_: effective diffusion coefficient in the cathode [m^2^/s]
*E*_OCV_: open-circuit voltage [V]
*F*: Faraday constant [96,485 C/mol]
h_*i*_^in^: specific enthalpy of species *i* at inlet [J/kg]
h_*i*_^out^: specific enthalpy of species *i* at outlet [J/kg]
*j*: current density [A/m^2^]
*j*_0_: exchange current density [A/m^2^]
LHV: lower heating value of the fuel [J/kg]
*ṁ*_*i*_^in^: mass flow rate of species *i* at inlet [kg/s]
*ṁ*_*i*_^out^: mass flow rate of species *i* at outlet [kg/s]
*ṁ*_fuel_: fuel mass flow rate [kg/s]
*ṅ*_CH_4_,anode_^in^: inlet molar flow rate of methane to the anode [mol/s]
*ṅ*_H_2__^in^: inlet molar flow rate of hydrogen [mol/s]
*ṅ*_CO_^in^: inlet molar flow rate of carbon monoxide [mol/s]
*NC*: number of chemical species or number of heaters [−]
*P*: system pressure [Pa]
*P*_H_2_O,_TPB__: partial pressure of H_2_O at TPB [Pa]
*P*_H_2,TPB__: partial pressure of H_2_ at TPB [Pa]
*P*_O_2,TPB__: partial pressure of O_2_ at TPB [Pa]
*P*_H_2_O_: partial pressure of H_2_O in the channel [Pa]
*P*_H_2__: partial pressure of H_2_ in the channel [Pa]
*P*_O_2__: partial pressure of O_2_ in the channel [Pa]
*Ṗ*_SOFC_: SOFC power output [W]
*Ṗ_j_*: power consumption of heater *j* [W]
*Q̇*: heat transfer rate [W]
*Q̇*_rev_: reversible heat generation [W]
*Q̇*_irr_: irreversible heat generation [W]
*Q̇*_ohm_: ohmic heating in the electrolyte [W]
*Q̇*_*i*,*k*_: heat supplied by the *i*-th hot stream in interval *k* [W]
*Q̇*_*j*, *k*_: heat required by the *j*-th cold stream in interval *k* [W]
*R_k_*: energy residue in interval *k* [W]
*R*_u_: universal gas constant [8.314 J/(mol ·K)]
*T*: temperature [K]
*U*_f_: fuel utilization factor [−]
*V*: operating voltage [V]
*V*_M*, i*_: model-predicted voltage at point *i* [V]
*V*_E*, i*_: experimental voltage at point *i* [V]
*ż*: fuel consumption rate [mol/s]
Δ*G*_act_: activation energy for ion migration [J/mol]
Δ*S*°: standard reaction entropy [J/(mol ·K)]
η_elec_: electrical efficiency [−]
η_act_: activation overpotential [V]
η_ohm_: ohmic overpotential [V]
η_conc_: concentration overpotential [V]
η_conc,a_: concentration overpotential at anode [V]
η_conc,c_: concentration overpotential at cathode [V]
ν: stoichiometric coefficient [−]
τ_electrolyte_: electrolyte thickness [m]
τ_anode_: anode thickness [m]
τ_cathode_: cathode thickness [m]
σ_electrolyte_: electrolyte conductivity [S/m]
ε*_i_*: relative error for test *i* [−]
ε: mean relative error [−]
