# Supplementary material for: Unraveling Heat Integration Opportunities in SOFC–Ethanol Reformer Systems across Steam Reforming, Partial Oxidation, and Autothermal Reforming Pathways
Source: ACS Omega. 2025 Nov 18;10(47):57724–40. doi: 10.1021/acsomega.5c09407 (PMC12676498; doi:10.1021/acsomega.5c09407)
Supplement: Supplementary file 1 [file ao5c09407_si_001.pdf]

Unraveling Heat Integration Opportunities in SOFC-Ethanol Reformer Systems Across Steam Reforming, Partial Oxidation, and Autothermal Reforming Pathways

Eduardo F Beathalter,[†] Guilherme P Pickler,[‡] Bruno F Oechsler,[¶] Amir A M Oliveira,[‡] and Rafael C. Catapan[*,§,‡]

†*Universidade Federal de Santa Catarina, 89219-600, Joinville, SC, Brazil*

‡*Graduate Program in Mechanical Engineering (POSMEC), Universidade Federal de Santa Catarina, 88040-900, Florianópolis, SC, Brazil*

¶*Graduate Program in Chemical Engineering (POSENQ), Universidade Federal de Santa Catarina, 88040-900, Florianópolis, SC, Brazil*

§*Graduate Program in Mechanical Science and Engineering (PPGECM), Universidade Federal de Santa Catarina, 89219-600, Joinville, SC, Brazil*

E-mail: rafael.catapan@ufsc.br

Supporting Information Available

Active area

The SOFC power output does not just correlate directly with the cell's power density, instead having a more intricate relation with the area, in this case, a scale-up strategy must be analyzed. Given that, the cell's power (Eq. 4 and 10) can be rewritten as a function

of the active area (A_c) and consumption molar rate ($\dot{z}$). As for low current densities the values of concentration losses are very small, it is possible to assume that $\eta_{conc} = 0$. The final equation is as follows:

$$P = 2F\dot{z}E_{OCV} - \frac{(2F\dot{z})^2}{A_c}\left(\frac{RT}{2Fj_0} + ASR\right) \tag{S1}$$

By taking the limit where the area tends to infinity, one can conclude that:

$$\lim_{A_c \to \infty} P(A_c) = 2F\dot{z}E_{OCV} \tag{S2}$$

Therefore, by equation S2 it is possible to conclude that an infinitely large fuel cell would have its power output dictated by the fuel flow rate and open circuit voltage. However, it is more useful to design a finite fuel cell that can actually be built. To do that, a selection criterion must be defined in order to specify the desired area. Let α be the ratio between the finite area fuel cell power output and the infinite area fuel cell power output. In mathematical notation:

$$\alpha \in [0, 1] \mid P\left(A_c\right) = \alpha P\left(\infty\right) \tag{S3}$$

Applying that to the equation S1, which can be rearranged and Isolating A_c:

$$A_c = \frac{2F\dot{z}\left(\frac{RT}{2Fj_0} + ASR\right)}{\left(1 - \alpha\right)E_{OCV}} \tag{S4}$$

Therefore, by using the equation S4 one can calculate the area necessary to reach an α fraction of the maximal power production for a given set of thermodynamic constraints and a specific fuel flow rate.

Table S1: Simulation results for five-factor DoE.

$H_2O/$ C_2H_5OH	$O_2/$ C_2H_5OH	P [atm]	T_{REF} [K]	T_{SOFC} [K]	$\dot{P}_{SOFC}$ [kW]	ES-HT-EW [kW]	ES-HT-AR [kW]	ES-REF [kW]	ES-HT-REF [kW]	ES-HT-AS [kW]	ES-SOFC [kW]	ES-CL-CATHODE [kW]	ES-CL-BUR [kW]
1	0	1	673	1023	0.966	1.091	0.000	-0.462	0.472	0.309	-0.242	-0.241	-9.144
3	0	1	673	1023	1.820	2.020	0.000	-0.316	0.702	0.650	-0.689	-0.507	-8.493
1	1	1	673	1023	1.534	1.091	0.434	-3.609	0.908	0.524	-0.503	-0.409	-6.187
3	1	1	673	1023	2.110	2.020	0.434	-3.513	1.137	0.794	-0.934	-0.619	-5.682
1	0	8	673	1023	0.383	1.091	0.000	-0.553	0.468	0.062	-0.072	-0.090	-12.089
3	0	8	673	1023	0.781	2.019	0.000	-0.497	0.694	0.133	-0.181	-0.193	-12.694
1	1	8	673	1023	0.637	1.091	0.032	-3.769	0.901	0.107	-0.137	-0.155	-8.925
3	1	8	673	1023	0.945	2.020	0.032	-3.725	1.127	0.165	-0.239	-0.240	-9.606
1	0	1	1073	1023	3.913	1.654	0.000	1.970	-0.076	4.689	-5.196	-4.202	-3.008
3	0	1	1073	1023	3.820	2.840	0.000	2.106	-0.112	5.194	-5.196	-4.707	-3.424
1	1	1	1073	1023	3.411	1.654	0.935	-1.966	-0.139	3.478	-2.570	-3.153	-3.082
3	1	1	1073	1023	3.337	2.840	0.935	-2.063	-0.174	3.481	-2.570	-3.156	-3.465
1	0	8	1073	1023	4.180	1.653	0.000	1.056	-0.075	1.641	-5.193	-2.562	-4.285
3	0	8	1073	1023	4.145	2.839	0.000	1.636	-0.112	2.328	-5.196	-3.839	-5.426
1	1	8	1073	1023	3.635	1.653	0.533	-2.128	-0.139	1.733	-2.560	-2.897	-4.247
3	1	8	1073	1023	3.578	2.839	0.533	-2.100	-0.174	1.831	-2.570	-3.078	-5.292
1	0	1	673	1123	0.973	1.092	0.000	-0.468	0.622	0.356	-0.208	-0.278	-9.299
3	0	1	673	1123	1.952	2.021	0.000	-0.324	0.923	0.747	-0.499	-0.583	-8.728
1	1	1	673	1123	1.600	1.092	0.435	-3.625	1.186	0.603	-0.385	-0.471	-6.454
3	1	1	673	1123	2.331	2.021	0.435	-3.513	1.485	0.912	-0.641	-0.712	-6.053
1	0	8	673	1123	0.373	1.091	0.000	-0.554	0.617	0.080	-0.072	-0.104	-12.243
3	0	8	673	1123	0.779	2.020	0.000	-0.498	0.913	0.171	-0.163	-0.223	-12.921
1	1	8	673	1123	0.630	1.091	0.032	-3.770	1.178	0.137	-0.128	-0.178	-9.208
3	1	8	673	1123	0.949	2.020	0.032	-3.725	1.473	0.210	-0.206	-0.275	-9.962
1	0	1	1073	1123	6.288	1.654	0.000	1.969	0.077	5.389	-2.676	-4.829	-3.223
3	0	1	1073	1123	6.186	2.840	0.000	2.106	0.113	5.969	-2.677	-5.409	-3.708
1	1	1	1073	1123	4.397	1.654	0.935	-1.965	0.140	3.997	-1.479	-3.623	-3.402
3	1	1	1073	1123	4.316	2.840	0.935	-2.038	0.176	4.000	-1.479	-3.627	-3.853
1	0	8	1073	1123	6.582	1.653	0.000	1.062	0.076	2.099	-2.676	-2.949	-4.497
3	0	8	1073	1123	6.543	2.839	0.000	1.636	0.113	2.931	-2.676	-4.340	-5.712
1	1	8	1073	1123	4.642	1.653	0.533	-2.098	0.140	2.249	-1.475	-3.388	-4.564
3	1	8	1073	1123	4.580	2.839	0.533	-2.099	0.176	2.339	-1.479	-3.537	-5.681
2	0.5	4.5	873	1073	4.830	1.884	0.211	-1.320	0.489	1.419	-2.568	-1.531	-4.453
3	0.5	4.5	873	1073	4.857	2.411	0.211	-1.207	0.558	1.664	-2.619	-1.864	-4.942

Continued on next page

Table S1 – *continued from previous page*

$H_2O/$ C_2H_5OH	$O_2/$ C_2H_5OH	P [atm]	T_{REF} [K]	T_{SOFC} [K]	$\dot{P}_{SOFC}$ [kW]	ES-HT-EW [kW]	ES-HT-AR [kW]	ES-REF [kW]	ES-HT-REF [kW]	ES-HT-AS [kW]	ES-SOFC [kW]	ES-CL-CATHODE [kW]	ES-CL-BUR [kW]
1	0.5	4.5	873	1073	4.160	1.358	0.211	-1.487	0.418	1.144	-1.827	-1.234	-5.069
2	1	4.5	873	1073	4.142	1.884	0.421	-2.901	0.615	1.525	-1.843	-1.760	-4.339
2	0	4.5	873	1073	4.310	1.884	0.000	0.137	0.362	1.194	-1.954	-1.289	-6.639
2	0.5	8	873	1073	4.098	1.884	0.139	-1.540	0.486	0.879	-1.758	-1.204	-6.490
2	0.5	1	873	1073	4.767	1.885	0.340	-0.658	0.495	3.371	-2.621	-2.935	-3.295
2	0.5	4.5	1073	1073	5.035	2.246	0.338	-0.029	0.000	3.224	-2.621	-4.022	-4.568
2	0.5	4.5	673	1073	0.931	1.556	0.088	-2.103	0.922	0.230	-0.206	-0.248	-9.467
2	0.5	4.5	873	1123	5.334	1.884	0.211	-1.342	0.615	1.556	-2.002	-1.638	-4.601
2	0.5	4.5	873	1023	3.768	1.884	0.211	-1.341	0.364	1.284	-3.690	-1.426	-4.300

$\boldsymbol{H_2O/C_2H_5OH}$: the water-to-ethanol molar ratio; $\boldsymbol{O_2/C_2H_5OH}$: oxygen-to-ethanol molar ratio; **P**: absolute pressure; $\boldsymbol{T_{REF}}$: reformer temperature; $\boldsymbol{T_{SOFC}}$: SOFC temperature; $\boldsymbol{\dot{P}_{SOFC}}$: SOFC eletrical power; **ES-HT-EW**: heat required to bring the water-to-ethanol mixture to the reformer temperature; **ES-HT-AR**: heat required to bring the air to the reformer temperature; **ES-REF**: reformer's heat balance; **ES-HT-REF**: heat required to bring the syngas to the SOFC temperature; **ES-HT-AS**: heat required to bring the air to the SOFC temperature; **ES-SOFC**: SOFC heat output; **ES-CL-CATHODE**: heat available in the cathode stream; **ES-CL-BUR**: heat available in the burner outlet stream.

Table S2: ANOVA results from the 5-factor central composite design. Q: quadratic, L: linear.

Factor	Sum-of-Squares Residual	F	p
H2O/C2H5OH(L)	0.000006	0.0025	0.960691
H2O/C2H5OH(Q)	0.000069	0.0269	0.870847
O2/C2H5OH(L)	0.001681	0.6561	0.423921
O2/C2H5OH(Q)	0.000527	0.2058	0.653180
P(L)	0.000017	0.0068	0.934844
P(Q)	0.000593	0.2316	0.633650
T_{REF}(L)	0.306358	119.5565	0.000000
T_{REF}(Q)	0.036022	14.0575	0.000704
T_{SOFC}(L)	0.019627	7.6596	0.009309
T_{SOFC}(Q)	0.000132	0.0514	0.822086

Table S3: Simulation results for two-factor DoE.

H_2O/C_2H_5OH	O_2/C_2H_5OH	P [atm]	T_{REF} [K]	T_{SOFC} [K]	$\dot{P}_{SOFC}$ [kW]	ES-HT-EW [kW]	ES-HT-AR [kW]	ES-REF [kW]	ES-HT-REF [kW]	ES-HT-AS [kW]	ES-SOFC [kW]	ES-CL-CATHODE [kW]	ES-CL-BUR [kW]
1	0	1	873	1073	5.208	1.358	0.000	0.547	0.294	2.279	-3.277	-1.778	-3.267
2	0	1	873	1073	5.351	1.885	0.000	0.817	0.368	3.049	-3.527	-2.525	-3.158
3	0	1	873	1073	5.337	2.411	0.000	1.064	0.440	3.654	-3.526	-3.130	-3.411
1	0.5	1	873	1073	4.779	1.358	0.340	-0.841	0.423	2.864	-2.621	-2.428	-3.052
2	0.5	1	873	1073	4.767	1.885	0.340	-0.657	0.495	3.371	-2.621	-2.935	-3.295
3	0.5	1	873	1073	4.744	2.411	0.340	-0.519	0.566	3.746	-2.620	-3.310	.3,532
1	1	1	873	1073	4.070	1.358	0.680	-2.423	0.550	2.985	-1.843	-2.636	-3.172
2	1	1	873	1073	4.045	1.885	0.680	-2.353	0.620	3.246	-1.842	-2.897	-3.399
3	1	1	873	1073	4.017	2.411	0.680	-2.312	0.690	3.416	-1.842	-3.067	-3.622

$\boldsymbol{H_2O/C_2H_5OH}$: the water-to-ethanol molar ratio; $\boldsymbol{O_2/C_2H_5OH}$: oxygen-to-ethanol molar ratio; **P**: absolute pressure; $\boldsymbol{T_{REF}}$: reformer temperature; $\boldsymbol{T_{SOFC}}$: SOFC temperature; $\boldsymbol{\dot{P}_{SOFC}}$: SOFC eletrical power; **ES-HT-EW**: heat required to bring the water-to-ethanol mixture to the reformer temperature; **ES-HT-AR**: heat required to bring the air to the reformer temperature; **ES-REF**: reformer's heat balance; **ES-HT-REF**: heat required to bring the syngas to the SOFC temperature; **ES-HT-AS**: heat required to bring the air to the SOFC temperature; **ES-SOFC**: SOFC heat output; **ES-CL-CATHODE**: heat available in the cathode stream; **ES-CL-BUR**: heat available in the burner outlet stream.

Table S4: Stream data from the flowsheet simulations assuming water-to-ethanol and oxygen-to-ethanol ratios as 1 and 0, respectivelly, $p = 1\ atm$, $T_{SOFC} = 1073\ K$ and $T_{REF} = 873\ K$.

	CP [kW/K]	T_{supply} [K]	T_{target} [K]	Description
C1	0.001652311	298.16	364.40	Water-ethanol mixture liquid state
C2	0.649703124	364.40	365.40	Water-ethanol mixture vaporization
C3	0.001176985	364.40	873.15	Water-ethanol mixture vapor state
C4	0.002939654	298.15	1073.15	SOFC air
C5	0.507883813	873.15	874.15	Reformer heat balance
C6	0.001471822	873.15	1073.15	Syngas
H1	0.001746296	1061.98	373.13	SOFC coolant vapor state
H2	1.887830231	374.13	373.13	SOFC coolant phase change
H3	0.003606725	373.13	323.15	SOFC coolant liquid state
H4	0.003365137	1344.82	373.15	Afterburner exhaust
H5	0.002294270	1073.15	298.15	SOFC cathode exhaust

Table S5: Heat cascade using $\Delta T = 10\ K$.

k	T1 [K]	T2 [K]	R_{k-1} [kW]	C1 [kW]	C2 [kW]	C3 [kW]	C4 [kW]	C5 [kW]	C6 [kW]	H1 [kW]	H2 [kW]	H3 [kW]	H4 [kW]	H5 [kW]	R_K [kW]
1	1339.83	1078.15	0.000000	0.000000	0.000000	0.000000	0.000000	0.000000	0.000000	0.000000	0.000000	0.000000	0.880582	0.000000	0.880582
2	1078.15	1068.15	0.880582	0.000000	0.000000	0.000000	0.029397	0.000000	0.014718	0.000000	0.000000	0.000000	0.033651	0.000000	0.870119
3	1068.15	1056.98	0.870119	0.000000	0.000000	0.000000	0.032825	0.000000	0.016435	0.000000	0.000000	0.000000	0.037576	0.025618	0.884053
4	1056.98	879.15	0.884053	0.000000	0.000000	0.000000	0.522770	0.000000	0.261740	0.310551	0.000000	0.000000	0.598435	0.407999	1.416528
5	879.15	878.15	1.416528	0.000000	0.000000	0.000000	0.002940	0.507884	0.001472	0.001746	0.000000	0.000000	0.003365	0.002294	0.911639
6	878.15	370.41	0.911639	0.000000	0.000000	0.597603	1.492581	0.000000	0.000000	0.886665	0.000000	0.000000	1.708616	1.164894	2.581629
7	370.41	369.41	2.581629	0.000000	0.649703	0.001177	0.002940	0.000000	0.000000	0.001746	0.000000	0.000000	0.003365	0.002294	1.935215
8	369.41	369.13	1.935215	0.000458	0.000000	0.000000	0.000815	0.000000	0.000000	0.000484	0.000000	0.000000	0.000933	0.000636	1.935995
9	369.13	368.15	1.935995	0.001623	0.000000	0.000000	0.002888	0.000000	0.000000	0.001715	1.854502	0.000000	0.003306	0.002254	3.793262
10	368.15	368.13	3.793262	0.000029	0.000000	0.000000	0.000052	0.000000	0.000000	0.000031	0.033328	0.000000	0.000000	0.000041	3.826580
11	368.13	318.15	3.826580	0.082586	0.000000	0.000000	0.146931	0.000000	0.000000	0.000000	0.000000	0.180273	0.000000	0.114673	3.892008
12	318.15	303.16	3.892008	0.024765	0.000000	0.000000	0.044060	0.000000	0.000000	0.000000	0.000000	0.000000	0.000000	0.034387	3.857570
13	303.16	303.15	3.857570	0.000000	0.000000	0.000000	0.000035	0.000000	0.000000	0.000000	0.000000	0.000000	0.000000	0.000027	3.857563
14	303.15	293.15	3.857563	0.000000	0.000000	0.000000	0.000000	0.000000	0.000000	0.000000	0.000000	0.000000	0.000000	0.022943	3.880505

Table S6: Comparison between simulated and calculated results.

Heat Exchanger	Cold Stream (CS)	CS Inlet Temperature [K]	CS Outlet Temperature [K]	Hot Stream (HS)	HS Inlet Temperature [K]	HS Outlet Temperature [K]	Exchanged Heat [kW]
1	C6	0.00%	0.00%	H4	0.01%	0.70%	0.00%
2	C5	0.00%	0.00%	H4	0.70%	1.85%	0.44%
3	C4	0.00%	0.00%	H4	1.85%	1.84%	-0.01%
4	CU	N/A	N/A	H4	1.84%	0.00%	-0.47%
5-7	C1-C3	0.00%	0.00%	H5	0.00%	1.94%	0.00%
8	CU	N/A	N/A	H5	1.94%	0.00%	-0.04%
9-11	CU	N/A	N/A	H1-H3	N/A	N/A	-0.01%
